# Environmental stress leads to genome streamlining in a widely distributed species of soil bacteria

**DOI:** 10.1038/s41396-021-01082-x

**Published:** 2021-08-18

**Authors:** Anna K. Simonsen

**Affiliations:** 1 Institute of Environment, Department of Biological Sciences, Florida International University, 11200 SW 8th Street, Miami, FL USA; 2 Centre of Excellence in Plant Energy Biology, Research School of Biology, Australian National University, 134 Linnaeus Way, Canberra, ACT Australia

**Keywords:** Population genetics, Microbial ecology, Bacterial genetics

## Abstract

Bacteria have highly flexible pangenomes, which are thought to facilitate evolutionary responses to environmental change, but the impacts of environmental stress on pangenome evolution remain unclear. Using a landscape pangenomics approach, I demonstrate that environmental stress leads to consistent, continuous reduction in genome content along four environmental stress gradients (acidity, aridity, heat, salinity) in naturally occurring populations of *Bradyrhizobium diazoefficiens* (widespread soil-dwelling plant mutualists). Using gene-level network and duplication functional traits to predict accessory gene distributions across environments, genes predicted to be superfluous are more likely lost in high stress, while genes with multi-functional roles are more likely retained. Genes with higher probabilities of being lost with stress contain significantly higher proportions of codons under strong purifying and positive selection. Gene loss is widespread across the entire genome, with high gene-retention hotspots in close spatial proximity to core genes, suggesting *Bradyrhizobium* has evolved to cluster essential-function genes (accessory genes with multifunctional roles and core genes) in discrete genomic regions, which may stabilise viability during genomic decay. In conclusion, pangenome evolution through genome streamlining are important evolutionary responses to environmental change. This raises questions about impacts of genome streamlining on the adaptive capacity of bacterial populations facing rapid environmental change.

## Introduction

A pervasive challenge in microbial ecology is detecting how natural microbe populations respond to environmental change. Prokaryotes have highly variable intraspecific genome content, described as a pangenome [1, 2]. Within a putative species cluster, all strains share a common set of genes (i.e. a ‘core genome’), while some genomic content is only present in a subset of strains (i.e. an accessory genome) [3]. Genome flexibility enables bacterial populations to rapidly respond to environmental change, and ecological adaptation has been invoked as a major process driving patterns of intraspecific pangenomic variation [4–9]. Compared to single nucleotide polymorphisms (SNPs), the acquisition or loss of whole genes through horizontal gene transfer has large potential to rapidly shift function and phenotype, and hence the strength of selection [2, 10]. This study shows how environmental stress leads to a consistent reduction in genome content in natural populations of a widely distributed bacteria species.

One approach in detecting changes in evolutionary pressures on genome evolution, such as ecological adaptation, is to determine how natural environmental variation, putative agents of natural selection, predict the distribution of genomic variation. Landscape genomics has been a powerful approach in uncovering the genetic basis of adaptation, traditionally detecting putative adaptive loci or SNPs on a single reference genome, and effectively concentrating adaptive discovery to the core genome of a species. However, little attention so far has been given to uncovering patterns of accessory genome variation across the environment [11]. Here, I apply a tailored landscape genomics approach to gain insight into the potential role of ecological variation in predicting accessory genome structure and composition, by examining how climate and soil related environmental factors shape the diversity and distribution of accessory genome content. This study focuses on an important type of genomic structural variation: variable presence of protein coding genes in the pangenome.

**Fig. 1:**
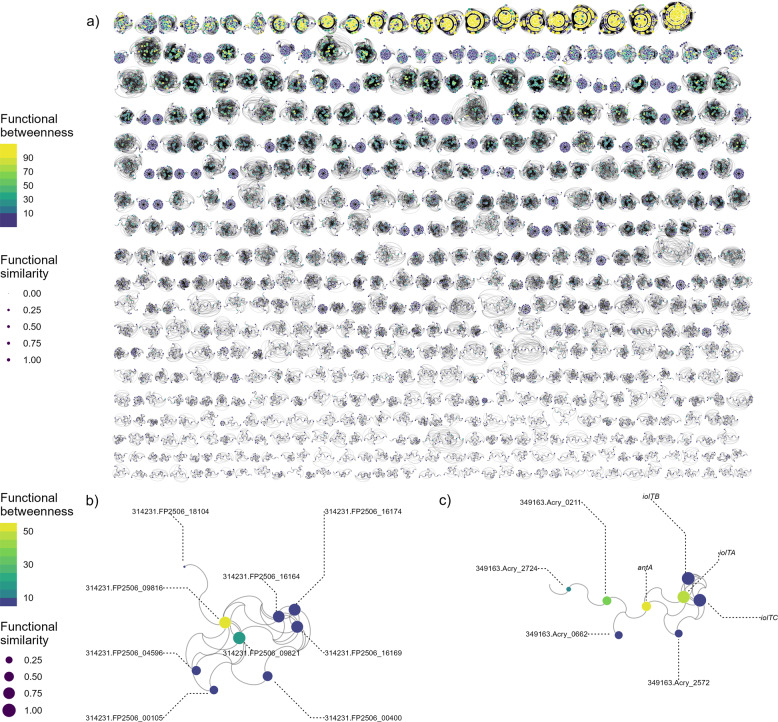
Gene interaction network for all annotated CDS regions. Each point corresponds to a different gene. **A** Entire network, except only subgraphs with greater than ten genes are plotted for visual clarity. Genes in the network but which were not found in the strains evaluated (i.e. ‘halo’ genes) are shown as edges ending with no dot. **B** and **C** Example subgraphs from the entire network with network traits rescaled. Labels indicate the preferred gene name according to eggNOG-mapper if available or the seed orthologue if not. An example of high betweenness can be seen with gene *antA* in **C**; removal of this gene would split two subnetworks. An example of genes with high similarity in **C** are) iolTA, iolTB, iolTC through their similarity in connections with other genes.

Genome reduction represents a major change in the accessory genome. Prokaryotic genomes are thought to be under constant decay due to a mutational bias towards deletion [12, 13]. One major prediction during genome reduction events is the loss of ‘superfluous’ or ‘non-essential’ genomic content [6, 13–18]. The function or feature of a genomic region is critical in predicting what genomic content is lost or retained during evolutionary shifts in genome content. Empirical tests have often focused on gene copies and pseudogenes (i.e. gene copies nearly identical in sequence similarity), which are predicted to be functionally redundant and more vulnerable to loss if they, for example, accrue a higher load of slightly deleterious mutations [12], or become inactivated during gene expression [13]. Beyond gene function based on sequence similarity, molecular genetic studies have revealed far more complex information on gene function, such as how proteins interact with other proteins to affect gene expression and phenotype, often conceptualised as a network [19]. For example, genes may have similar functions because they have very similar network connections with other proteins (Fig. 1b,c), and removing one of these genes is predicted to have small or negligible negative impact on fitness. In contrast, genes may also hold central functions by linking numerous gene regulatory pathways together, and so removing these genes would potentially rearrange networks of other essential functions and have a large negative impact on fitness (Fig. 1b,c). Therefore, gene network properties potentially provide a useful framework to predict changes in genomic content during genome reduction.

Using a landscape genomic approach, this study investigates how four environmental stress gradients (temperature, rainfall, soil pH and soil salinity; Fig. S1) have shaped accessory genome content in natural populations of a widespread soil bacterial species with a large genome, *Bradyrhizobium diazoefficiens*, which was previously sampled across a contiguous and environmentally complex heterogeneous landscape [20] (see Method A). *Bradyrhizobium diazoefficiens* provide critical ecosystem services through symbiotic nitrogen-fixation with legume host roots (a.k.a rhizobia). Prior to symbiosis, rhizobia colonies must survive and adapt to free-living conditions, and evolutionary responses to variable free-living climate and soil stressors is the focus here. To determine the role of environmental stress as a putative agent of selection in driving pangenome evolution, I examine accessory gene loss patterns to detect environment-specific signals of gene loss as well as functional (network and duplication traits) and molecular (strength of purifying and positive selection) gene properties that predict gene loss and retention. This study further examines how chromosomal structural properties and population differentiation patterns in the core genome may be contributing to environmentally stress-induced accessory gene loss and retention patterns.

## Materials and methods

### A. Strain sampling and isolation

*Bradyrhizobium* is a commonly occurring genus in soil [21]. Closely related *Bradyrhizobium diazoefficiens* (previously *Bradyrhizobium japonicum*) strains were isolated from soil, as previously described [20, 22]. In brief, *Bradyrhizobium* isolates that formed symbiotic associations with a foundational legume species in the censused region, *Acacia acuminata*, were isolated from soil sampled along a large region spanning ~300,000 km^2^ in South West Australia, a globally significant biodiversity hotspot [23]. In total 60 soil samples were collected from twenty sites (3 soil samples per site; Supplementary Fig. S1) and 380 isolates were sequenced (19 isolates per site, 5 or 6 isolates per soil sample, each isolate re-plated from a single colony at least 2 times). Host *A. acuminata* legume plants were inoculated with field soil in controlled chamber conditions and isolates were cultured on Mannitol Yeast agar plates from root nodules (see [20, 22] for details). A total of 374 strains were included in this study after removing 5 contaminated samples and one sample that was a different *Bradyrhizobium* species; non- *Bradyrhizobium diazoefficiens* sample removal was determined from 16S rRNA sequences extracted from draft genome assemblies (Method C) using RNAmmer [24].

### B. Environmental variation among sampled sites

In this study, I focus on environmental factors (temperature, rainfall, soil pH and salinity) previously identified to impact either rhizobia growth performance, functional fitness or persistence in soil [25–28] and where a directionality of rhizobial stress response could be attributed with respect to environmental variation present in the sampled region (i.e. stress occurs at high temperatures, low rainfall, high acidity and high salinity). Each environmental factor was standardised to a mean of 0 and a standard deviation of 1, and pH and rainfall scales were reversed to standardise stress responses directions so that low stress is at low values and high stress is at high values for all factors (Supplementary Fig. S2). Additionally, salinity was transformed using a log transformation (log(*x* + 0.01) to account for some zeroes) prior to standardisation.

### C. Isolate sequencing and pangenome annotation

Illumina short reads (150 bp paired-end) were obtained and draft genome assemblies were generated using Unicycler from a previous study [29]. Resulting assemblies were of good assembly quality (99.2% of all strains had >95.0% genome completeness score according to BUSCO [30]; Table S1; assembled using reads that contained nominal 0.016 ± 0.00524% non-prokaryotic DNA content across all 374 isolates, according to Kraken classification [31]). Protein coding regions (CDS regions) were identified using Prokka [32] and assembled into a draft pangenome using ROARY [33], which produced a matrix of counts of orthologous gene clusters (i.e. here cluster refers to a set of protein-coding sequences containing all orthologous variants from all the different strains, grouped together and designated as a single putative gene). Gene clusters that occurred in 99% of strains were designated as ‘core genes’ and used to calculate the ‘efficiency of selection’ [34, 35] (measured as dN/dS, Method G.2) and population divergence measured as Fixation Index ‘Fst’, Method H) across each environmental stress factor. The identified gene clusters were then annotated using eggNOG-mapper V2 [36] and the strain by gene cluster matrix was reaggregated using the Seed ortholog ID returned by eggNOG-mapper as the protein identity. Out of the total 2,744,533 CDS regions identified in the full sample of 374 strains, eggNOG-mapper was able to assign 2,612,345 of them to 91,230 unique Seed orthologs. These 91,230 protein coding genes constituted the final protein dataset for subsequent analyses.

### D. Calculation and statistical analysis of gene richness and pangenome diversity along the stress gradient

Gene richness was calculated as the total number of unique seed orthologues for each strain (i.e. genome). Any singleton genes that occurred in only a single strain, as well as ‘core’ genes that occurred in every strain (for symmetry, and because these are equally uninformative with respect to variation between strains) were removed, leaving 74,089 genes in this analysis. Gene richness (being count data) was modelled on a negative binomial distribution (glmer.nb function) as a function of each of the four environmental stressors as predictors using the lme4 package in R [37], also accounting for hierarchical structure in the data by including site and soil sample as random effects.

To rule out potentially spurious effects of assembly quality (i.e. missed gene annotations due to incomplete draft genomes) on key findings, I confirmed no significant association between gene richness and genome completeness (*r* = 0.042, *p* = 0.4224, Fig. S3).

Finally, pangenome diversity was calculated as the total number of unique genes that occurred in each soil sample (since multiple strains were isolated from a single soil sample). Pangenome diversity was modelled the same as gene richness, except here soil sample was not included as a random effect.

### E. Calculation of network and duplication traits for each gene

I used the seed orthologue identifier from eggNOG-mapper annotations to query matching genes within StringDB ([38]; https://string-db.org/), which collects information on protein-protein interactions. Out of 91,230 query seed orthologues, 73,126 (~80%) returned a match in STRING. For matching seed orthologue hits, a network was created by connecting any proteins that were annotated as having pairwise interactions in the STRING database using the igraph package in R [39]. Two vertex-based network metrics were calculated for each gene: betweenness centrality, which measures a genes tendency to connect other genes in the gene network, and mean cosine similarity, which is a measure of how much a gene’s links to other genes are similar to other genes.

Betweenness centrality was calculated using igraph (functional betweenness). For mean cosine similarity, a pairwise cosine similarity was first calculated between all genes. To do this, the igraph network object was converted into a (naturally sparse yet large) adjacency matrix and the cosSparse function in qlcMatrix in R [40] was used to calculate cosine similarity between all pairs of genes. To obtain an overall cosine similarity trait value for each gene, the average pairwise cosine similarity to all other genes in the network was calculated.

Finally, gene duplication level was calculated for each gene as one additional measure of ‘redundancy’, by calculating the average number of gene duplicates found within the same strain. Duplicates were identified as CDS regions with the same Seed orthologue ID.

### F. Gene distribution models

To determine how gene traits predict accessory genome distributions patterns along the stress gradients, I first calculated a model-based metric (hereafter and more specifically a standardised coefficient, ‘*z*-score’) of the relative tendency of each gene to be found in different soil samples across the four stress gradients (heat, salinity, acidity, and aridity). This was achieved by modelling each gene’s presence or absence in a strain as a function of the four stress gradients, with site and soil sample as a random effect, using a binomial model in lme4 (the structure of the model being the same as the gene richness model, only the response is different). To reduce computational overhead, these models were only run for the set of genes that were used in the gene richness analysis (e.g. after removing singletons and core genes), and which had matching network traits calculated (e.g. they occurred in the STRING database; *n* = 64,867 genes). Distribution models were run in tandem for each gene using the manyany function in the R package mvabund [41]. Standardised coefficients, or *z*-scores (coefficient/standard error) indicating the change in the probability of occurrence for each gene across each of the stress gradients were extracted. More negative coefficients correspond to genes that are more likely to be absent in high stress (and vice versa for positive coefficients).

To determine how network and duplication traits influence the distribution of genes across the stress gradient, I performed a subsequent linear regression model where the gene’s *z*-scores was the response and gene traits as predictors. The environmental stress type (i.e. acidity, aridity, heat and salinity) was included as a categorical predictor, and the interaction between stress category and gene function traits were used to infer the influence of gene function traits on gene distributions in a given stress type (see Supplementary Methods S1 for z-score transformation).

### G. Quantifying molecular signals of natural selection on accessory and core genes

To examine molecular signatures of selection in accessory and core genes, I calculated dN/dS for a subsample of the total pool (*n*=74,089 genes), which estimates the efficiency of selection [34, 35]. Two major questions relevant to dN/dS that are addressed here require a different gene subsampling approach:

#### (1) Do variable environmental stress responses lead to different dN/dS patterns among accessory genes?

Here, I subsampled accessory genes (total accessory gene pool across 374 strains, 74,089) to generate and compare dN/dS among 3 categorical groups, each representing a different level of stress response based on their z-scores (accessory genes that either strongly increase, decrease or have no change in occurrence as stress increases; *n* = 1000 genes/category; see Supplementary Methods S2 for subsample stratification details).

For each gene, sequences were aligned using codon-aware alignment tool, MACSE v2 [42]. dN/dS was estimated by codon within each gene using Genomegamap’s Bayesian model-based approach [43], which is a phylogeny-free method optimised for within bacterial species dN/dS calculation (see Supplementary Methods S3 for dN/dS calculation/summarisation; S9 for xml configuration). The proportion of codons with dN/dS that were credibly less than 1 (purifying selection) and those credibly greater than 1 (positive selection) were analysed, with respect to the genes’ occurrence response to stress. Specifically, I modelled the proportion of codons with dN/dS < 1 using a beta regression (suitable for response data expressed as a proportion), with the stress response category as a predictor. The proportion of codons with dN/dS > 1 was overall too low to analyse in this way, so the binary outcome (a gene with any codons with dN/dS > 1 or not) was modelled using a binomial response model with the response categories as predictors (see Supplementary Methods S4 for details of both models).

#### (2) Does dN/dS among microbial populations vary across environmental stress?

Here, I compared the average change in dN/dS in core genes present across all environments at the population level (i.e. all isolates from one soil sample), which is used here to measure the change in the efficiency of selection across each stress gradient. Core genes were used since they occur in all soil samples, allowing a consistent set and sample size of genes to be used in the population-level dN/dS calculation. This contrasts with the previous section, which quantifies gene-level dN/dS on extant accessory genes that intrinsically have variable presence or absence across soil samples. For computational feasibility, 500 core genes were subsampled (total core 1015 genes) and, for each gene, individual strain variants were collated into a single fasta file based on soil sample membership, such that dN/dS could be calculated separately for each gene within each soil sample (i.e. 60 soil samples × 500 genes = 30,000 fasta files). Each fasta file was then aligned in MACSE and dN/dS calculated using the same methodology for accessory genes (Supplementary Method S3). This enabled the average dN/dS in a sample to be associated with soil-sample level environmental stress variables. Specifically, I modelled the mean proportion of codons with dN/dS < 1 in a soil sample (where the mean was taken over all genes in the soil sample), as a linear function of the sample’s four environmental stress variables in a multiple beta regression (see Supplementary Methods S5 for model details). There was insufficient power to analyse the proportion of codons with dN/dS > 1 due to overall rarity of positive selection (average proportion of genes where at least 1 codon with dN/dS > 1 was ~0.006). This low level of positive selection is expected for core genes which tend to be under strong selective constraint.

### H. Calculation and analysis of Fixation index (Fst) along stress gradients

Using the core genome alignment (all SNPs among 1015 core genes) generated previously with ROARY, I computed pairwise environmentally-stratified Fst. Each soil sample (*n* = 60) was first placed into one of 5 bins based on their distances in total environmental stress space (using all four stress gradients), with the overall goal of generating roughly evenly sized bins such that the environmental similarity of stress was greater within bins than between (see Supplementary Methods S6 and Fig. S4 for clustering algorithm details). Next, fasta alignments were converted to binary SNPs using the adegenet package. Pairwise Fst between all strains (originating from a particular soil sample) within a single bin was calculated using StAMPP in R [44]. For each strain pair, the average of the two stress gradient values was assigned.

The relationship between pairwise Fst and the average stress value was evaluated using a linear regression model with each of the four stress values as predictors. Since the analysis uses pairwise data (thus violating standard independence assumptions), the significance of the relationship was determined using a permutation test (see Supplementary Methods S7 for details).

### I. Chromosomal structure analysis of gene loss patterns

To gain insight into structural variation and test for regional hotspots in gene loss along the chromosome, I mapped each gene’s stress response (i.e. probability of loss or gain indicated by each genes *z*-score) onto a completed *Bradyrhizobium* genome (strain ‘36_1’ from the same set of 374 strains (Genbank CP067102.1; [45]). Putative CDS positions on the complete genome were determined using Prokka and annotated with SEED orthologue ID’s using eggNOG-mapper. Matching accessory genes derived from the full set of 374 incomplete draft genomes (*n* = 74,089) were mapped to positions on the complete genome (6274 matches). The magnitude of gene loss or gain (as measured by their standardised *z*-scores for each environment from the gene distribution models; see Method F) was then modelled across the genome using a one-dimensional spatial smoothing model. This model was implemented in R INLA [46] (www.r-inla.org), and models a response in a one-dimensional space using a Matern covariance-based random effect. The method uses an integrated nested Laplace approximation to a Bayesian posterior distribution, with a cyclical coordinate system to accommodate circular genomes. The model accounts for spatial non-independence among sites and produces a continuous posterior distribution of average z-score predictions along the entire genome, which was then used to visualise potential ‘hotspots’ of gene loss or gain. The modelling procedure was repeated, instead with gene network traits, such that model predictions of similarity and betweenness could be visualised on the reference chromosome.

## Results

### *Bradyrhizobium* strains have highly variable accessory genomes

Pangenomic variation has been documented in numerous bacterial species across terrestrial, marine, aquatic and atmospheric systems [2, 47], and as expected there is also considerable accessory genome variation across all *B. diazoefficiens* isolates. Consistent with previous work [48], all isolates share a set of genes in the core genome, but many genes occur at variable frequencies across all isolates (Fig. S5), indicating large compositional turn-over in accessory genome content. The mean number of genes per genome (hereafter ‘gene richness’; see Method C and D) was also variable, ranging from 5641 to 8734 (5603–8220 with singletons removed), and was expectedly highly correlated with estimated genome size (Fig. S6; average genome size 7.78 Mbp).

### There is pervasive gene loss along an environmental stress gradient

**Fig. 2:**
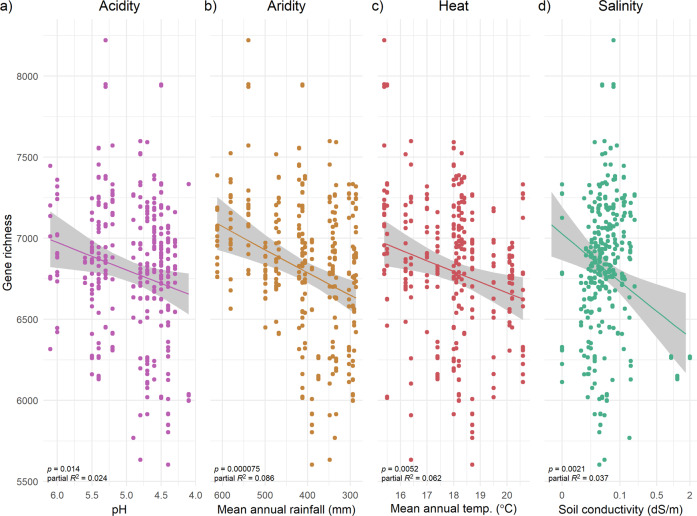
Gene richness (total number of unique seed orthologues/strain) significantly decreases as environmental stress increases across four gradients (A acidity, B aridity, C heat, D salinity). Fitted lines (with 95% CI) show model predictions after accounting for environmental collinearity in a multiple regression model (see Method D). *P* values from Table S2 model results are shown, along with partial *R*^2^ measures (see Supplementary Methods S10). Each data point is a strain (*n* = 374). Raw gene richness values are plotted. Singleton accessory genes and core genes are removed from the gene richness count, *n* = 74,089 genes.

**Fig. 3:**
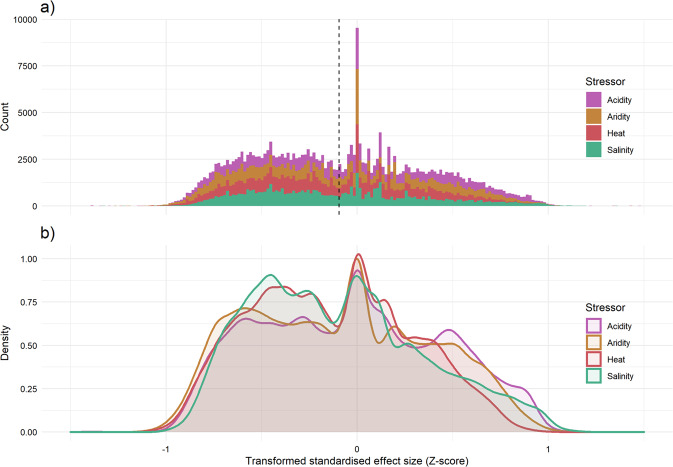
Histogram plot of raw z-score values derived from gene distribution models. A negative *z*-score indicates that genes have a lower probability of occurrence as stress increases, while a positive *z*-score indicates that genes have a higher probability of occurrence in high stress (see Method F). **A** Histogram of z-scores **B** Density plot of z-scores, drawn using a smoothed kernel density estimate. All mean *z*-scores for each environmental stress are statistically different from zero (*p* < 0.0001, see main effects in Table S3). Across all four environmental stresses there is an enrichment of negative *z*-scores (especially in heat and salinity; see main effect model estimates in Table S3), indicating a tendency for gene loss along all gradients and also showing a qualitative consistency of results between gene-level and strain-level model analyses (Table S2). (*n* = 74,089 ge*n*es).

All evaluated environmental factors strongly predicted gene richness (total number of genes/genome, Fig. 2; Table S2), and all specifically show that strains isolated from higher stress environments consistently have fewer genes in their genome (on average 336, 458, 342 and 674 fewer genes for acidity, aridity, heat and salinity stress, respectively per genome). Additional independent analyses further validated the robustness of the genome reduction trend. Firstly, the predictive power of environmental stress on gene richness was recapitulated using different gene annotation pipelines: a database-dependent method (eggNOG-mapper) and an orthologous gene clustering method (Table S2). Secondly, when the distribution of genes was modelled individually as a function of their environmental predictors (see Method F), all genes overall have a higher probability of being absent in more stressful conditions across all 4 environmental factors (Fig. 3), recapitulating gene loss patterns observed at the strain level (Fig. 2).

These analyses show that genes in the accessory genome, on average, have a higher chance of being lost in environments of high stress, or have a higher chance of being retained in low stress environments. Because gene richness is highly correlated with genome size (Fig. S6), these results strongly suggest that environmental stress causes, on average, ~0.5 million base pair reduction in bacterial genome size across all four stress gradients, mediated (at least impart) by overall losses in protein-coding genes. These results provide a clear indication of differential evolutionary pressures on pangenome content being driven by different soil and climate conditions. Given the strong pattern of observed gene loss, in the next 2 sections I demonstrate what genes have a higher probability of being lost and retained based on their functional properties.

### Gene network properties predict environmental patterns of genome reduction

**Fig. 4:**
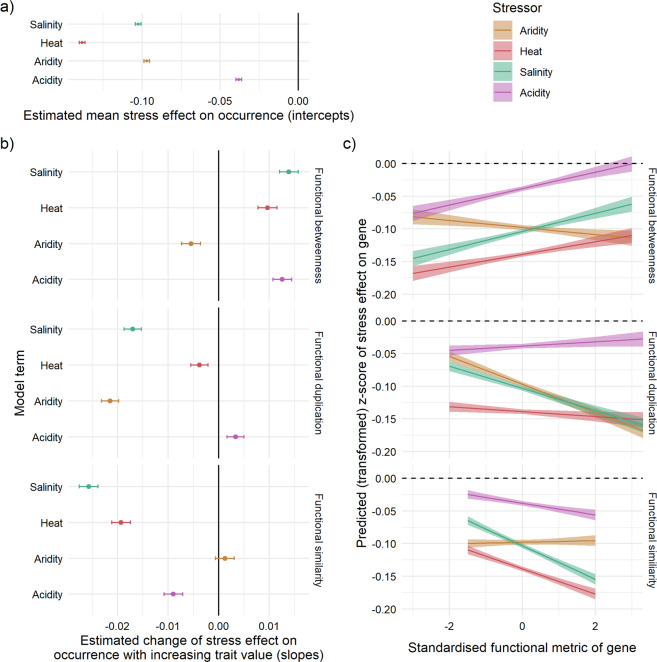
Effect of gene functional traits (similarity, betweeness and duplication) on a gene’s stress response (z-score). Network and duplication gene traits were incorporated into the gene-level distribution model as predictors to determine whether and to what extent gene functional traits modify how each environmental factor predicts gene loss along the stress gradient (a graphical representation of Table S3). All points in **A** and **B** whose error bars (95% confidence intervals) do not overlap zero are statistically significant (*p* < 0.05; see Table S3) a mean stress effects are all negative coefficients indicative of an overall tendency towards gene loss b) Interaction term coefficients between the gene trait and the environmental stress factor and **C** predicted z-score of stress effect based on main and interaction model terms (95% confidence interval is shown). Functional Betweenness and Similarity are derived from gene interaction networks, while duplication is derived from genome annotations. All environmental factors on average support the hypothesis that genes which are more functionally redundant (high Functional Similarity and Duplication) are more likely to be lost (negative slope), while multi-functional genes (high functional Betweenness) are more likely to be retained (positive slope) in high stress.

A global protein-protein network of *B. diazoefficiens* from a curated database (STRING, https://string-db.org/) was leveraged to compute two major network statistics for each gene: mean cosine similarity and betweenness-centrality from the pangenome network (Fig. 1b; see Method E). Genes with a high mean cosine similarity have, on average, a higher similarity of connections to other genes, and are predicted to have a minimal effect on fitness in the event of a gene loss due to a putative availability of a functional replacement. In contrast, betweenness measures the degree to which a gene acts as a ‘bridge’ or link to other gene networks so genes with high betweenness are predicted to have a large effect on fitness due to their large potential to disrupt linkages between other gene networks or clusters (Fig. 1b). I found that genes with a high mean cosine similarity value have a significantly higher probability of being absent in high stress environments while genes with high betweenness have a low probability of being absent in higher stress (Fig. 4b; Table S3). In other words, genes that show high connection similarities with other genes tend to become lost in higher stress (Fig. 4b; Table S3). Genes with high betweenness have a higher probability of being retained (i.e. the same probability across the whole stress gradient) or increase in high stress environments (Fig. 4b; Table S3). However, aridity affects gene network properties differently from other environmental factors: either showing no effect of cosine similarity or comparatively a much smaller effect size in the opposing direction for betweenness (Fig. 4b; Table S3).

Together these analyses show, at least for three (heat, pH, salinity) of four environmental stress gradients evaluated here, that network trait values describing how gene products interact with each other at a molecular level, have informative properties that allow us to predict which genes may be more vulnerable to loss (i.e. a higher cosine similarity), or which genes may be more resistant to pervasive decay (i.e. high betweenness) as an evolutionary response to environmental stress.

### Gene duplicates are more easily lost during genome reduction

Genes that have duplicate copies in each genome tended to have lower probability of occurrence in more stressful environments, the exception being acidity with a weaker effect size in the opposing direction to other environmental factors (Fig. 4; Table S3). While aridity had weak predictive power of gene loss through network traits (Fig. 4; Table S3), in contrast aridity strongly affected the predictive power of gene duplicates, nearly double the effect size of the other environmental factors (see Fig. 4). These results again support the hypothesis that genes which are functionally redundant have a higher probability of being lost, along similar lines of interpretation with cosine-similarity, generally consistent with previous findings [49]. While network traits were highly predictive for heat, pH, salinity, gene duplicate traits are highly predictive for the remaining factor (aridity but also salinity) demonstrating support for the overall role of functional redundancy in predicting gene loss across all four environmental factors. There was no evidence of environment-induced gene loss (or gain) attributable to COG, a widely utilised categorisation of gene function (Table S4; Fig. S7).

### Genes involved in genome streamlining show strong molecular signatures of selection

**Fig. 5:**
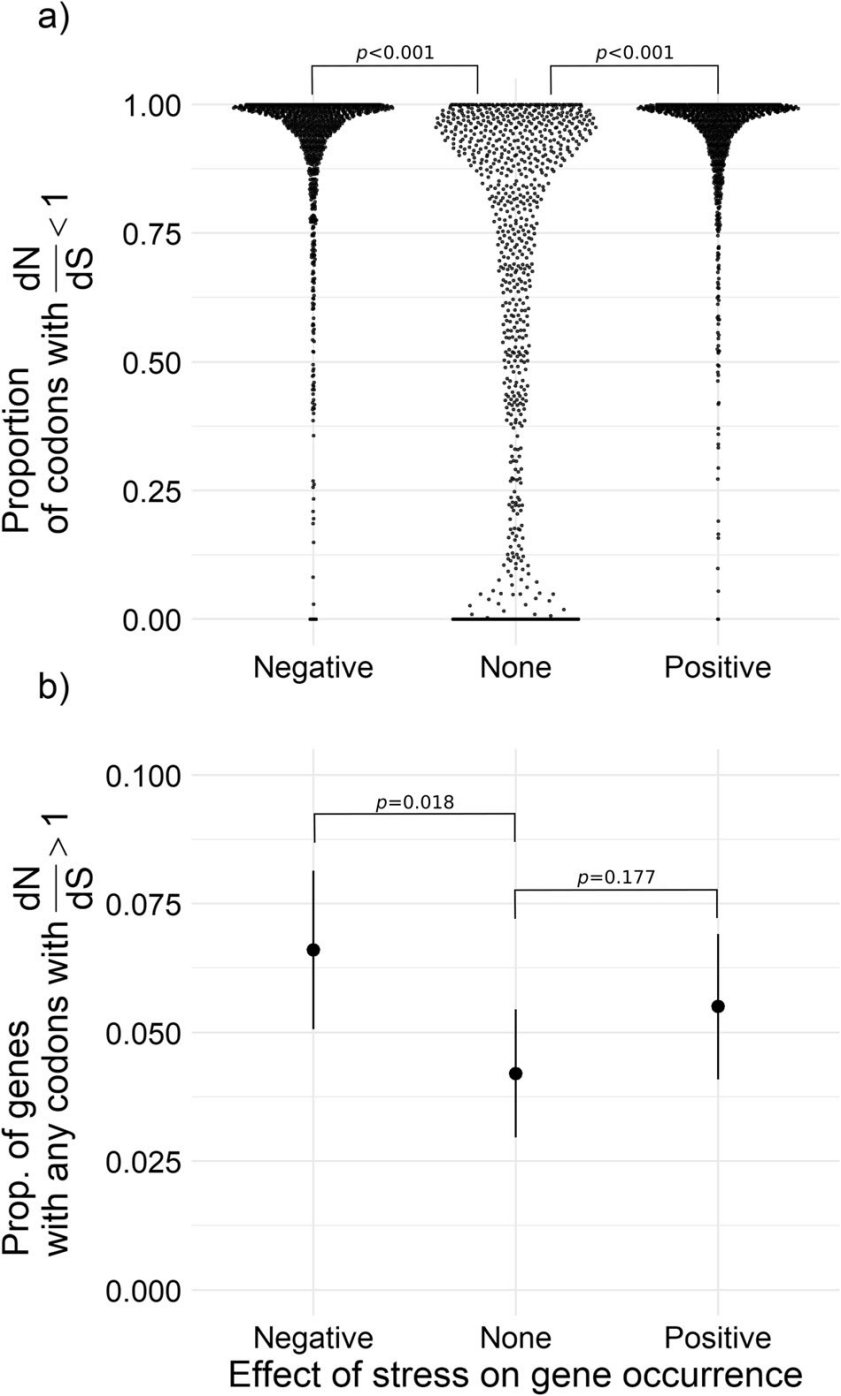
Influence of environmental stress on dN/dS in stratified subsample of accessory genes in pangenome. **A** Each point shows proportion of codons with dN/dS values < 1 (purifying selection) within a single accessory gene. Genes are categorised based on their stress response (*z*-score, based on occurrence along the stress gradient; *n* = 3000 genes, 1000 per category). Genes with small or no responses to stress (*z*-score ~ 0) overall have a lower proportion of codons under purifying selection (*p* < 0.001; Table S5). **B** Points show the mean and 95% prediction interval for the proportion of genes with any codons with dN/dS values > 1 (positive selection), plotted by each occurrence response category, based on a binomial model. Accessory genes with negative stress response have significantly higher proportion of genes under positive selection (*p* = 0.018; Table S5).

Expectedly, purifying selection (dN/dS credibly < 1) was dominant in protein-coding accessory genes, with only a small proportion of codons under positive selection (on average 5.6% of codons/gene). I found that accessory genes that are prone to loss in high stress (*z*-score < 1) have a significantly higher percentage of codons either under purifying selection (dN/dS < 1; Fig. 5a; Table S5 for statistical model results) or positive selection (dN/dS credibly > 1; Fig. 5b; Table S5), compared to accessory genes that neither had a tendency for gain or loss (*z*-score ~ 0). While genes gained with increasing stress (i.e. genes that tend to only occur in higher stress environments; strongly positive *z*-score) also had a significantly higher percentage of codons under purifying selection, they showed no difference for codons under positive selection (Fig. 5; Table S5) compared to accessory genes that neither had a tendency for gain or loss (*z*-score ~ 0).

The key findings here are that accessory genes that respond to environmental stress through their environmental occurrence show distinct molecular signatures of selection, where extant strain level variants of these accessory genes in the population appear to be under strong purifying selection, regardless of the direction of response to stress. Furthermore, accessory genes that most strongly contribute to the observed genome reduction patterns (i.e. genes with a higher probability of being lost with increasing stress), tend to harbour more codons under both strongly purifying and positive selection.

### The pattern of gene loss and efficiency of selection is environmentally dependent

Given the consistent pattern of gene loss along all four environmental stresses (Fig. 2), I further tested if the type of environmental stress leads to differences in the pattern of selection that is potentially mediating gene loss.

**Fig. 6:**
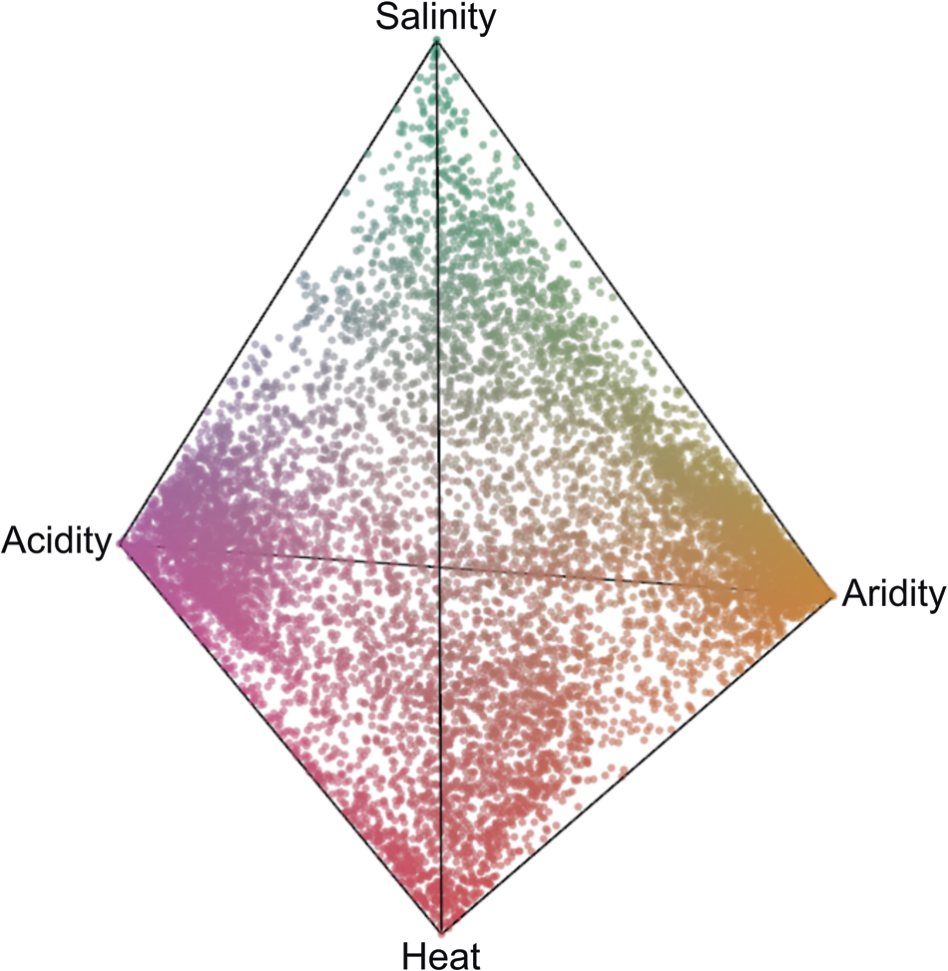
3-D tetrahedron showing weighting of gene loss on each environmental stress factor based on z-scores derived from gene distribution models. There is an enrichment of genes exclusively lost in either highly acid or arid soils. Each data point is a single gene and only genes with strong propensities to become lost with at least one increasing stress gradient are shown (*z*-score < −2, Supplementary Methods S8). Points at or near tetrahedron tips show genes with a large relative weighting of *z*-scores to one specific environmental factor while points between tips indicate genes that have z-scores with more similar values in at least two factors. Points on or near the centre of the tetrahedron show genes that have small or no inequalities among all four *z*-scores. Environment-specific weighting of gene loss is also indicated by the colour. Points near the triangle tips are colour coded [acidity = pink, aridity = orange, heat = red and salinity = green]. Points with colour blending indicate genes more likely to be lost in multiple environments. An interactive 3-dimensional mode of this plot is available as a supplementary file.

Firstly, I hypothesised that particular genes may be disadvantageous and selectively removed in specific environmental conditions (see Supplementary Methods S8 for details). Consistent with this hypothesis, a clear pattern of environment-specific gene loss was found (Fig. 6, see Supplementary Information for an interactive version of Fig. 6); specifically, a higher enrichment of genes that are exclusively lost in either highly arid and acidic environments. However, for salinity and heat stress, there is a much weaker signal of environment-specific gene loss (Fig. 6). Consistently, there is also less drastic reductions in pangenome diversity (i.e. mean gene richness among all strains within a soil sample) with increasing acidity and aridity compared to heat and salinity (Table S2), providing further evidence of a more targeted gene-specific pattern of loss in the accessory genome that is environmentally specific.

Secondly, I tested if the type of environmental stress alters the change in the efficiency of selection as each stress gradient increases. Environmental stress has strong potential to simultaneously change selective pressures and effective population size, which together rebalance the net interplay between selection and drift, shifting the efficiency of selection (measured as dN/dS, the ratio of non-synonymous to synonymous substitutions, see Method G), where a lower selection efficiency (dN/dS values closer to 1) indicates weaker selection and/or smaller effective population size [12, 50]. Since effective population size is often interpreted at the population level, dN/dS values here were calculated in core genes to enable robust population-level dN/dS comparisons across all environments (see Method G.2, as oppose to the previous results section which calculates dN/dS statistics on accessory genes at the gene-level). As expected for core protein-coding genes, an overwhelming proportion of codons per gene were under purifying selection (>99.9% dN/dS credibly less than 1), indicating that the potential range of change in the efficiency of selection is 0 < dN/dS < 1. When comparing the mean patterns of change in dN/dS in core genes that were present in all environments, I found that the proportion of codons under strong purifying selection significantly decreases as heat and salinity stress increase but showed no change with acidity and aridity stress (Table S6). This strongly supports the conclusion that an increase in stress decreases the efficiency of selection (at least in the core genome), but that this observed decrease is context dependent, only occurring as heat and salinity stress increases. Comparison of Fixation Indices (Fst) across all environmental gradients shows congruent patterns, where microbial populations differentiation significantly increases with stress, but only with increasing heat and salinity (Table S7; Fig. S8).

Together, these analyses indicate that although all four environmental stresses lead to genome reduction, there is a strong indication that the type of stress leads to different evolutionary pressures and processes that may be driving gene loss outcomes, which are demonstrated here by the contrast in dynamics between acidity and aridity versus heat and salinity in core and accessory genes. While there is suggestive evidence of a more targeted environmentally-specific accessory gene selection as acidity and aridity stress increase, the evidence presented here also show that the efficiency of selection becomes weaker as heat and salinity stress increase.

### Accessory gene retention during stream-lining has chromosomal structure that coincides with core gene location

**Fig. 7:**
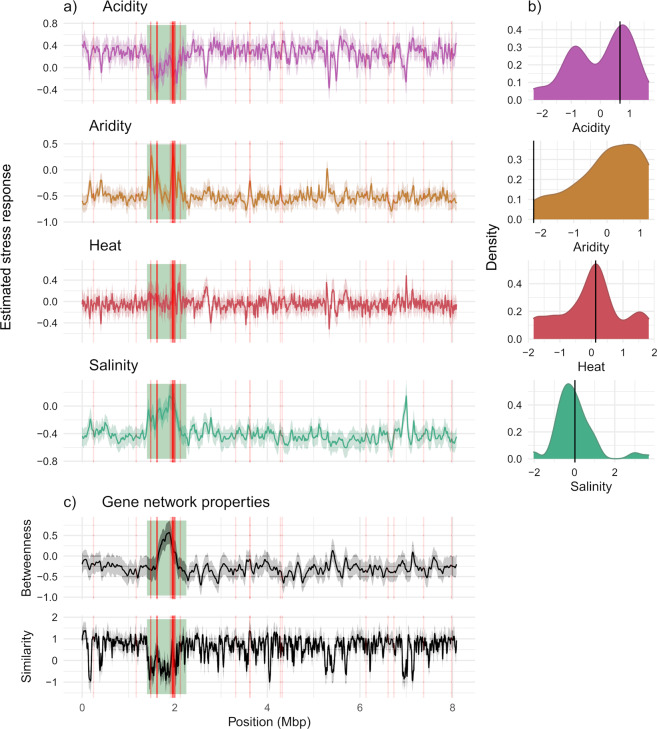
Chromosomal mapping of gene loss/gain patterns and functional network trait values on complete genome sequence. 6274 out of 74,089 accessory genes in the pangenome could be matched and mapped to genome 36_1 (strain 36_1 from the same 374-strain population set, Genbank accession CP067102.1; Method I). **A** and **C** Together show that prominent changes in stress response and network trait properties concentrate in a large ~850 kbp region (green box), which also has a higher density of mapped core genes (red lines) along the chromosome. Solid lines (other than vertical red) are model prediction values that account for spatial non-independence in genomic position, while faded surrounding colours indicates 95% credibility. **A** Mapping of gene loss and gain patterns, based on gene occurrence stress responses (i.e. gene *z*-scores) with 95% credibility. **B** Smoothed data distribution of each environmental stress factor. Solid vertical line indicates stress values of soil sample where reference genome 36_1 originated. **C** Chromosomal mapping of gene network properties, similarity and betweenness. Notes on interpreting **A**: regions of gene-retention are indicated by values that change towards 0, regardless of whether the predominant *z*-score value is highly positive or negative. The predominant z-score is influenced by the sample of matching orthologue genes and the environmental origin of the reference genome. For example, in **A** acidity, the predominant z-score is strongly positive as a statistical expectation because the strain originates from more acidic environments. Whereas for aridity, the predominant z-score is negative because the strain originates from a relatively wetter environment.

Finally, I tested the hypothesis that gene loss occurs in ‘hotspot’ or ‘island’ locations in the *Bradyrhizobium* genome. Using a complete genome reference from one of the 374 strains examined in this study, gene loss was instead generally found to be widespread across the genome with hotspots of low gene loss (Fig. 7a). Consistent with network trait findings of gene loss patterns, accessory genes with higher betweenness tend to occur within these gene-retention hotspots, while accessory genes with high network similarity are widespread (Fig. 7c). These regions of low gene loss tend to co-locate where a large concentration of core genes occur and spatially cluster in one major ~850 kbp region (Fig. 7a,c). These results demonstrate for this particular reference strain, that patterns of genome decay through loss of protein coding regions can follow distinct positional structure along the genome that is also likely to be mediated by spatial proximity to core genome regions.

## Discussion

This study provides strong evidence that environmental conditions (climate and soil) dictate patterns of accessory genome evolution in natural populations of a free-living soil bacteria species, *Bradyrhizobium diazoefficiens*, which also undergo facultative symbiosis with legumes. Here, I have demonstrated a clear pattern of genome reduction, through the loss of protein coding genes along four environmental stress gradients (see Table 1 for key results summary). Specifically, bacterial strains isolated from hotter, drier, more acidic and more saline soils show reduced genome size, showing strong support for the hypothesis that ‘superfluous’ genes (predicted either by gene duplication or similarity in gene protein networks) have a higher probability of being lost during genome reduction in all stress gradients. Furthermore, this study shows that genes more likely to have multi-functional roles (i.e. act as bridges of multiple gene networks)—and predicted to be more essential—are more likely to be retained during genome reduction (along most environmental gradients).

**Table 1:** Summary of key results.

	Increasing acidity	Increasing aridity	Increasing heat	Increasing salinity
Gene richness trend	4.8% gene loss	6.5% gene loss	4.9% gene loss	9.5% gene loss
Functional Gene Properties	Higher loss in genes with similar protein-protein interaction networks	Higher loss in gene duplicates	Higher loss in genes with similar protein-protein interaction networks	Higher loss in gene duplicates and genes with similar protein-protein interaction networks
Environment-specific gene loss?	Yes, strong signal of acidity-specific gene loss	Yes, strong signal of aridity-specific gene loss	Weaker signal of heat-specific gene loss	Weaker signal of salinity-specific gene loss
Molecular selection signatures on stress-responsive accessory genes	Strong purifying and positive selection	Strong purifying and positive selection	Strong purifying and positive selection	Strong purifying and positive selection
Change in efficiency of purifying selection in core genes	No change	No change	Decreasing efficiency	Decreasing efficiency
Core genome population differentiation (Fst)	Decreasing differentiation (trending non-significantly)	Decreasing differentiation (trending non-significantly)	Increasing differentiation	Increasing differentiation

Genome reduction or ‘streamlining’ has been observed in numerous bacterial species. The most striking examples of extreme reductions in genome size are during speciation transitions from free-living to obligate and environmentally stable host conditions, where genes have been lost due to drift because their cellular functions have been outsourced to the host in obligate symbiosis [51, 52]. Extreme reductions in genome size have also been observed in free-living microbial species [14, 53], due to selection driven streamlining [16, 54, 55]. Insights on genome reduction within species have come from single-strain time-series experiments in controlled and environmentally homogenous laboratory settings [6, 17, 56], but also highlight the methodological challenge of replicating ecologically relevant environments in laboratory settings. Here, by examining microevolutionary changes of the pangenome within a bacterial species (having both symbiosis and free-living life-history) along numerous environmental clines, this study demonstrates the importance of ecological context in driving different evolutionary pathways of genome streamlining and demonstrates how large and diverse the accessory component of bacterial pangenomes are in naturally occurring populations. Evaluating naturally recombining bacterial populations along ecologically-relevant environmental gradients also reveals that gene loss is widespread across the genome with islands of low gene loss that co-locate near and among core genes. Core genes are predicted to provide essential functions required for cell viability and reproduction [57], and the results suggest that *Bradyrhizobium* (at least in one strain) has evolved a spatial arrangement of core genome structure (i.e. clustered instead of dispersed) that may more likely be preserved in the event of any genome reduction processes.

There is a wide debate on the extent to which changes in accessory genome are influenced by neutral or adaptive processes [7, 11, 58, 59] with two contrasting theoretical explanations on why bacterial genomes streamline: (1) Redundant gene loss is neutral and genome decay is due primarily due to drift, where effective population size determines what genes are perceived as nearly neutral, combined with a mutational bias towards deletion [13, 58] (2) Redundant genes are lost primarily because bacterial genomes are persistently fine-tuning to minimise cellular inefficiency through selection [12, 15, 16]. The fact that two seemingly opposite evolutionary forces, selection and drift, can be predicted to have the same effect on gene loss, depending on the assumptions made, underscores the conceptual challenge of distinguishing the causes of genetic patterns in wild populations. Despite this, the combined evidence from this study suggests that while both drift and selection likely co-contribute to higher gene loss in stressful conditions, their relative balance differs depending on the type of environmental stress that is acting.

First of all, there is strong evidence that selection, in general, is playing a role in accessory genes whose occurrence responds to environmental stress. I found that accessory genes which are strongly responding to stress (i.e. through their occurrences in different sampling locations across environmental gradients) are under stronger selective pressure, having significantly higher proportion of codons under purifying or positive selection compared to accessory genes randomly distributed with respect to environmental stress. Because accessory genes that only occur in extreme ends of the stress gradient (i.e. either in high stress [large positive *z*-score] or low stress [large negative *z*-score]) appear to be under strong selection (especially strong purifying selection; Fig. 5), this generally suggests that selection is maintaining the presence of these accessory genes in their respective environmental extremities. However, because molecular signatures (such as dN/dS) can only be measured in existing accessory gene variants in the population and not in those that are missing (e.g. ‘lost’), this limitation strongly merits future theoretical development that is beyond the scope of this study. Despite this limitation, the data presented here demonstrates that stress-responsive accessory genes show distinct molecular signatures of selection and suggests that selection at the whole-gene level and molecular selection on the sequences within individual genes are correlated. When synthesising all of the following evidence together—(a) the environmental stress gradients evaluated here are known to affect rhizobia function and fitness, (b) the same stress gradients affect accessory genes through their distribution patterns (whole gene presence/absence), and (c) the stress-associated distribution patterns are strongly coincident with molecular selection at the codon level—it all jointly supports the hypothesis that selection is playing a role in shaping pangenome evolutionary responses to climate and soil-associated environmental stress.

Second, although selection appears to have a general role in the accessory component of the pangenome, there is some suggestive evidence that its strength relative to drift in explaining gene loss patterns varies by the type of stress. Specifically, higher heat and salinity appear to create distinct population genetic patterns, as indicated by a decrease in the efficiency of selection (dN/dS) and increase in population differentiation (Fst) with high stress in core genes, when compared to aridity and acidity. The observed decrease in the efficiency of selection provides strong evidence of either weaker selection and/or smaller effective population size in the core genome at high heat and salinity stress. Consistent with this study’s finding, theoretical models predict that increased population differentiation (Fst) can be a result of decreased effective population size through increased population sub-division [60]. The contrast in environment-specific evolutionary response was also observed by the fact that accessory genes that had a higher probability of loss in aridity and acidity also had a stronger tendency to be ‘uniquely lost’ along these two stress gradients, rather than ‘co-lost’ along all stresses, which was more common for genes with high loss in heat and salinity (Fig. 6). If we make the theoretical assumption that the observed reduction in the efficiency of selection is a genome-wide effect (as a result of reduced effective population size) that also affects the accessory genes, this study’s findings suggest that heat and salinity stress causes more stochastic loss of genes that cannot overcome the drift barrier [60]. Future studies will be required to disentangle the differential responses of heat and salinity versus acidity and aridity observed here, and to provide theoretical frameworks that mechanistically link coevolutionary changes in core and accessory genes. Likewise, effective population size remains a persistent technical challenge to address in future studies, especially in free-living soil bacteria for targeted species that co-occur with hundreds of other taxonomically related species in the soil. Regardless of the evolutionary mechanism causing genome reduction, this study clearly shows that environmental stress not only plays an important role in determining the composition of accessory genes in the pangenome, but also the patterns of molecular evolution in both core and accessory genes.

The pervasive genome decay along soil and climate-related stress gradients also has implications regarding evolvability in bacterial populations. The observed loss of genetic diversity (at the gene level) with increasing stress prompts future questions and experiments on the upper bounds of genome decay that microbial populations can tolerate with increasing environmental stress. The loss of genetic diversity also raises questions about how genome streamlining impacts the ability for bacterial populations to evolutionarily respond to environmental change when already faced with stressful conditions.

## Supplementary information

Supplemental Information

Supplementary interactive 3D version of Figure 6

## Data Availability

All sequence data for this study have been deposited in PRJNA669073 SRA archives at NCBI. R scripts are available upon request.
