# Supplementary material for: Environmental stress leads to genome streamlining in a widely distributed species of soil bacteria
Source: ISME J. 2021 Aug 18;16(2):423–34. doi: 10.1038/s41396-021-01082-x (PMC8776746; doi:10.1038/s41396-021-01082-x)
Supplement: Supplementary file 1 — Supplemental Information [file 41396_2021_1082_MOESM1_ESM.pdf]

Supplementary Information

Supplementary Methods

Methods S1: z-score transformation details

To determine how network and duplication traits influence the distribution of genes across the stress gradient, I performed four subsequent models where the gene's z-scores (for a given stress factor) was the response and gene traits as predictors. Prior to analysis I transformed the model-based metrics to reduce the extremity of the tails of their distribution and thus better meet the normality, linearity, and homogeneity of variance assumptions of linear regression analysis. Since the z-scores could be both negative and positive, and appear to have long tails in both directions, I used the recommended bi-symmetric log transformation [1]. The transformation is as follows (default value of C suggested in [1], $C = 1/\log(10)$:

$$\mathrm{biSymLog}\,(x) = \mathrm{sign}\,(x)\log_{10}\left(1 + |\frac{x}{C}|\right)$$

Methods S2: Stratified subsampling of accessory genes

To calculate dN/dS among the 3 major stress response categories, I first performed a stratified subsampling based on each gene's z-score value. Subsampling was done because calculating dN/dS on 74,089 genes in the total pangenome set was not computationally feasible. Genes that occur in less than 10 strains were removed from the selection pool in order to have sufficient sample size to calculate dN/dS per gene. These categories were: 1) genes with little response to stress: chosen as the top 1000 genes with the maximum absolute z-score closest to zero, 2) genes that were strongly suppressed by stress (losses): chosen as the top 1000 genes with the lowest minimum z-score (strong negative values), and 3) genes that were strongly promoted by stress (gains): chosen as the top 1000 genes with the highest maximum z-score (strong positive values). For all chosen genes, all strain level sequence variants of each gene were collated into individual gene fasta files (3000 fasta files) for downstream alignment (in MACSE) and dN/dS calculation in Genomegamap.

Methods S3: dN/dS calculation and summarisation

To calculate dN/dS, which here estimates the efficiency of selection [2, 3], I used a Bayesian model-based approach implemented in Genomegamap [4], which is robust to within-species recombination. I calculated dN/dS on a per codon basis using the 'sliding window' model, which models each codon's dN/dS value as distributed in spatial 'blocks' along the genes, where the number and distribution of blocks is estimated by the model using a 'reversible jump' MCMC algorithm. I used standard relatively uninformative priors for all parameters in each model (lambda = unif(), dN/dS[i] = unif(), etc.). I ran the MCMC chain on each gene for 100,000 iterations, discarded the first 20,000 iterations as burn-in and thinned the remaining samples to every 100, resulting in 800 total samples from the posterior for subsequent analysis. See Supplementary S9 for example Genomegamap xml configuration.

I used the posterior distribution of codon-wise dN/dS estimates to summarise information on sequence-level selection as follows. For each gene I counted how many codons had posterior distributions that did not overlap 1 with 95% credibility, dividing them into those greater than 1 (positive selection), and those less than 1 (purifying selection). I used this to calculate the proportion of codons with dN/dS < 1, and those with dN/dS > 1 within each gene and used these as a response variable.

Methods S4: Beta regression model for analysis of dN/dS among subsampled accessory genes

To analyse the proportion of codons with dN/dS that were credibly < 1 (purifying selection), I implemented a beta regression model. Examination of the distribution of values showed a bimodal distribution with enrichment of values near 0 and near 1 (most genes had nearly all codons < 1, or nearly none); this data distribution is suitable to be analysed with a beta distributed error structure (beta regression). I used *betareg* in R [5] to model the mean proportion of codons in a gene having an estimated dN/dS < 1 as a function of model predictors (3 stress response categorical groups). The category of genes with no response to stress was chosen as the reference level, so the estimates and p-values associated with the negative and positive response to stress categories reflect their difference from the no response category. To analyse the probability of a gene containing at least 1 positive dN/dS codon, I implemented a binomial model, with the predictors exactly the same as the beta regression model.

Methods S5: Beta regression model for analysis of dN/dS among subsampled core genes

For the core gene analysis I analysed estimates of the proportion of codons with dN/dS < 1 across soil samples (Supplementary Methods S3). For each core gene, an estimate was made using an alignment containing only the variants of each gene found in a particular soil sample (see Methods G main text). As with dN/dS values for accessory genes, the data has a beta distributed error structure. In order to implement a beta regression in *betareg* in R, I collapsed the data down to one observation per soil sample by taking the mean proportion of codons with dN/dS less than 1 across all 500 genes found within each soil sample. This could then be analysed using the environmental stress values calculated for each soil sample as predictors. An examination of the residuals of a model with all four stress variables as predictors showed non-homogeneity of variance, which appeared to be associated with the stress variables. Beta regression allows the variance component of the beta distribution to be modelled also as a function of covariates. To account for non-homogeneity of variance I modelled variance as a linear model of all four stress variable predictors. After accounting for changes in variance across stress, model residuals showed good correspondence with model assumptions of homogeneity of variance. Subsequently I only interpret the change in the mean proportions with respect to stress, but the model coefficients describing the change in variance of the proportions with stress is also reported in the results Table S6.

Method S6: Soil sample clustering algorithm

The goal of this step was to assign soil samples into bins based on environmental similarity, clustering a minimum of 11 soil samples per bin to ensure relatively even cluster membership. This binning was achieved by the following steps: 1) Soil samples were hierarchically clustered according to their stress gradient values using he “Ward D” method [6] in *hclust* in R. 2) An iterative binning approach (“bottom-leaves” hierarchical clustering; a modified approach of [7]) was used to achieve approximately even (as possible) membership number within each bin or cluster. Specifically, I used *cuttree* (on an iterative sliding scale in increments of h=0.0001) to find the first cut point that identified the first environmentally similar cluster (11 members in size) in the hierarchy. This cluster was assigned as the first bin and removed from the hierarchy. To find the next environmentally similar cluster membership bin (11 members in size), I used a sliding-scale *cuttree* again on the reduced hierarchy and then removed the associated members from the hierarchy, as in the previous step. This procedure was repeated until soil samples could be assigned to all 5 bins (Figure S4), providing roughly evenly sized bins such that the environmental similarity of stress was greater within bins than between.

Method S7: Permutation test to detect change in Fst with stress

To determine the four environmental factors significantly predicted a change in Fst as stress increases, I implemented a permutation test due to the model violating standard assumptions of non-independence). For 500 replications I randomly shuffled the rows and columns of the pairwise Fst matrix between all soil samples and recalculated the pairwise Fst values (using the same bins as above). I reran the same linear regression as above instead using the reshuffled Fst values as the response. This produced a null distribution of regression coefficients that we expect under a completely randomized scenario to compare to the observed coefficients, and allowed me to implement tests of significance for each stress factor identical to a standard Mantel test.

Methods S8: Visualising tendency towards loss in different stress gradients at the gene-level

I used visualisation techniques to identify environment-specific gene loss patterns across the stress gradient, specifically, based on barycentric coordinates within a three dimensional tetrahedron (a three dimensional version of the standard ternary or 'de Finetti' plot [8]). I plotted genes within a tetrahedron such that their distance to the four corners, which represented the four stress gradients, was proportional to their relative tendency to be lost in each stress gradient (i.e. the degree of inequality of gene loss along all four stress gradients). I filtered genes to a subset that had at least one negative z-score (i.e. coefficient) less than -2 (i.e. 2 standard errors from 0). I then inverted each z-score by multiplying them by -1, such that values corresponding to greater loss would be higher. Each genes' four inverted z-scores were then transformed to a simplex (e.g. values between 0 and 1 that sum to 1) using a softmax transformation[9], which would be used as barycentric coordinates with respect to the four tetrahedron corners. Barycentric coordinates (in 4-D, one dimension for each stress gradient) were transformed to cartesian coordinates in 3-dimensional space using the bary2cart function in the geometry package in R. Finally, genes were plotted as points using the *rgl* package [10] for 3d visualisation.

Method S9: Genomegamap example XML template

```
<?xml version="1.1"?>
<!-- genomegaMap Bayesian inference sliding window model example  -->
<!-- This template is set up to infer variation in dN/dS (omega) along the gene -->
<gcat xmlns="http://www.danielwilson.me.uk/gcat">
        <libraries>
                <!-- Location of the genomegaMap shared object (dynamic library) -->
                <library file="libgenomegaMap.so"/>
        </libraries>

        <data>
                <!-- Location of the FASTA-format sequences. Can also be specified as codon frequencies: see XML reference
-->
                <codon_count id="seqs" distribution="seqs~" file=
"{fasta_alignment}"/>
        </data>

        <parameters>
                <!-- Definitions of parameters and their initial values -->
                <!-- Bayesian inference requires prior distributions for parameters -->
                <!-- Diversity parameter -->
                <continuous_scalar id="theta" distribution="theta~" value="0.17"/>
                <!-- Transition:transversion ratio -->
                <continuous_scalar id="kappa" distribution="kappa~" value="1.0"/>
                <!-- dN/dS ratio -->
                <!-- This is a mosaic (piecewise constant vector) initialized with a single boundary -->
                <continuous_mosaic id="omega" distribution="omega~" length="seqs" boundaries="0" values="1"/>
                <!-- Codon frequencies (excluding STOP codons) in the order
                        TTT TTC TTA TTG TCT TCC TCA TCG TAT TAC TGT TGC TGG CTT CTC CTA CTG CCT CCC CCA CCG CAT CAC CAA
CAG CGT CGC CGA CGG ATT ATC ATA ATG ACT ACC ACA ACG AAT AAC AAA AAG AGT AGC AGA AGG GTT GTC GTA GTG GCT GCC GCA GCG GAT
GAC GAA GAG GGT GGC GGA GGG
                 -->
                <continuous_vector id="pi">
                        0.0163934426229508 0.0163934426229508 0.0163934426229508 0.0163934426229508 0.0163934426229508
0.0163934426229508 0.0163934426229508 0.0163934426229508 0.0163934426229508 0.0163934426229508 0.0163934426229508
0.0163934426229508 0.0163934426229508 0.0163934426229508 0.0163934426229508 0.0163934426229508 0.0163934426229508
0.0163934426229508 0.0163934426229508 0.0163934426229508 0.0163934426229508 0.0163934426229508 0.0163934426229508
0.0163934426229508 0.0163934426229508 0.0163934426229508 0.0163934426229508 0.0163934426229508 0.0163934426229508
0.0163934426229508 0.0163934426229508 0.0163934426229508 0.0163934426229508 0.0163934426229508 0.0163934426229508
0.0163934426229508 0.0163934426229508 0.0163934426229508 0.0163934426229508 0.0163934426229508 0.0163934426229508
0.0163934426229508 0.0163934426229508 0.0163934426229508 0.0163934426229508 0.0163934426229508 0.0163934426229508
0.0163934426229508 0.0163934426229508 0.0163934426229508 0.0163934426229508 0.0163934426229508 0.0163934426229508
0.0163934426229508 0.0163934426229508 0.0163934426229508 0.0163934426229508 0.0163934426229508 0.0163934426229508
0.0163934426229508 0.0163934426229508
                </continuous_vector>
        </parameters>

        <transformations>
                <!-- Specify the NY98 mutation model rate matrices for the alignment -->
                <ny98_pdrm id="mut" theta="theta" kappa="kappa" omega="omega" pi="pi" length="seqs"/>
                <!-- Extract the number of omega blocks along the alignment -->
                <continuous_mosaic_num_blocks id="nblo" continuous_mosaic="omega"/>
        </transformations>

        <distributions>
                <!-- Priors -->
                <!-- Improper priors are specified for theta and kappa -->
                <improper_log_uniform_distribution id="theta~"/>
                <improper_log_uniform_distribution id="kappa~"/>
                <!-- The prior on omega is specified in two steps and cannot be improper -->
                <gamma_distribution id="marginal_omega~" shape="1" scale="1"/>
                <continuous_mosaic_distribution id="omega~" p="{p}" marginal="marginal_omega~"/>
                <!-- Likelihood function for genomegaMap -->
```

```
		<genomegaMap id="seqs~" mut="mut"/>
	</distributions>

	<mcmc niter="{mcmc_niter}" seed="timer" screen_update="1">
		<!-- Metropolis Hastings proposals for the parameters -->
		<log_uniform_proposal parameter="theta" half-width="{theta_halfwidth}" weight="1"/>
		<log_uniform_proposal parameter="kappa" half-width="{kappa_halfwidth}" weight="1"/>
		<continuous_mosaic_log_uniform_proposal parameter="omega" half-width="1" weight="{block}"/>
		<!-- Reversible jump proposals for changing the omega block boundaries -->
		<continuous_mosaic_extend_block parameter="omega" mean_extension="10" weight="{block}"/>
		<continuous_mosaic_splitmerge_block parameter="omega" p="{p}" weight="{block}" mean_type="geometric"/>

		<log burnin="0" thinning="{thinning}" file="{mcmc_output_file}">
			<!-- Parameters to be output -->
			<parameter idref="theta"/>
			<parameter idref="kappa"/>
			<parameter idref="omega"/>
			<parameter idref="nblo"/>
			<!-- Log conditional probability masses/densities to be output -->
			<loglikelihood idref="seqs"/>
		</log>
	</mcmc>
</gcat>
```

Method S10: Partial pseudo-R^2 measure reported in Figure 2

The measure of variance explained by each fixed variable was calculated for the gene richness model (Method D) using the R package rsq [11, 12], which produces partial pseudo-R^2 measures for mixed effects (both fixed and random effects part of the model). I calculated partial R^2 for the total fixed effects of the full model and with each stress variable removed. The difference between the full fixed effect R^2 for the full and reduced model is a measure of partial R^2 for the particular variable removed. In order to accommodate multicollinearity between the stress variables, I calculated the partial R^2 for each variable when included in the model with all combinations of the other stress variables, and reported the mean partial R^2 over these models [13]. All partial pseudo-R^2 measures for each variable sum to the total R^2 for the fixed effects of the model ($R^2 = 0.20$).

Supplementary Tables

Table S1: Mean and correlations (*p*-values) between assembly statistics and genome completeness (BUSCO %). Correlations indicate that genomes with higher completeness expectedly tend to have better assembly quality (higher N50 and lower L50).

			Correlations (N50 and L50 logged)		
Statistic	Median	SD	Completeness	N50	L50
Completeness	99.8	1.043	— (p=—)	0.313 (*p*=0.000)	−0.292 (*p*=0.000)
N50	202,205.0	130,430.953	0.313 (*p*=0.000)	— (*p*=—)	−0.945 (*p*=0.000)
L50	12.0	11.111	−0.292 (*p*=0.000)	−0.945 (*p*=0.000)	— (*p*=—)

Table S2. Effect of four environmental stresses gradients on gene richness (# unique genes/genome, n=374 strains) and pangenome diversity (# unique genes/microbial population). Here, microbial population constitutes a single soil sample (n=60 soil samples). Gene richness is calculated using seed orthologue ID as the gene identifier based on annotations from eggNOG-mapper (Method C and D main text). For additional validation, gene richness was also calculated based on the number of gene ortholog clusters identified de novo from ROARY, and similar results were found. All measures show consistent reduction of gene richness as stress increases. Pangenome diversity significantly reduces in heat and salinity gradients only. Shown below are fixed and random effects of several mixed models (negative binomial distribution for all models). R^2 values calculated using the rsq package in R (Zhang, 2021). *p*-values are in brackets.

	**Gene Richness (SEED orthologs)**	**Gene Richness (Roary)**
Intercept	8.824***	8.899***
	(0.000)	(0.000)
Aridity	**-0.019*** **	**-0.023*** **
	**(0.000)**	**(0.000)**
Heat	**-0.014*** **	**-0.016*** **
	**(0.005)**	**(0.002)**
Salinity	**-0.017*** **	**-0.019*** **
	**(0.002)**	**(0.001)**
Acidity	**-0.012** **	**-0.014*** **
	**(0.014)**	**(0.010)**
Soil Sample RE SD	0.026	0.031
Site RE SD	0.009	0.004
AIC	5489.8	5611.9
BIC	5521.2	5643.3
R^2 - Fixed effects	0.20	0.21
R^2 - Total	0.39	0.41
Log.Lik.	-2736.901	-2797.942

* $p < 0.1$, ** $p < 0.05$, *** $p < 0.01$

Table S2 (continued).

	**Pangenome Diversity**
Intercept	9.631***
	(0.000)
Aridity	-0.057*
	(0.075)
Heat	**-0.099*** **
	**(0.002)**
Salinity	**-0.173*** **
	**(0.000)**
Acidity	-0.035
	(0.290)

* p < 0.1, ** p < 0.05, *** p < 0.01

Table S3. Effect of gene functional traits (similarity, betweeness and duplication) on a gene's stress response (as measured by its z-score from the gene distribution model). The z-score was transformed to reduce skew (see Supplementary Method S1) and then analysed with a linear regression model which included the type of stress (Acidity, Aridity, Heat, or Salinity) as a categorical variable, and the interaction between the type of environmental stress and three gene functional redundancy traits: The average number of copies of a gene per genome and two network measures based on a gene-gene interaction network (see Method E main text). Betweenness measures how often a gene is an intermediary between other genes in the interaction network, whereas similarity measures how similar a gene's links in the interaction network are, on average, to other genes that are present in the sample. The model therefore estimates the relationship or slope between each gene's functional trait value and a gene's stress response (z-score), separately for each type of stress along with a stress type specific intercept, here referred to as the stress type 'Main Effect').

	Estimate (p-value)
Acidity Main Effect	**-0.038 (0.000)*** **
Aridity Main Effect	**-0.097 (0.000)*** **
Heat Main Effect	**-0.138 (0.000)*** **
Salinity Main Effect	**-0.103 (0.000)*** **
Acidity × Betweenness	**0.013 (0.000)*** **
Aridity × Betweenness	**-0.005 (0.003)*** **
Heat × Betweenness	**0.010 (0.000)*** **
Salinity × Betweenness	**0.014 (0.000)*** **
Acidity × Similarity	**-0.009 (0.000)*** **
Aridity × Similarity	0.001 (0.503)
Heat × Similarity	**-0.019 (0.000)*** **
Salinity × Similarity	**-0.026 (0.000)*** **
Acidity × Copy Number	**0.003 (0.050)** **
Aridity × Copy Number	**-0.021 (0.000)*** **
Heat × Copy Number	**-0.004 (0.026)** **
Salinity × Copy Number	**-0.017 (0.000)*** **
Num.Obs.	247188
R^2	0.049
R^2 Adj.	0.049
AIC	317790.8
BIC	317967.9
Log.Lik.	-158878.404
F	796.790

* $p < 0.1$, ** $p < 0.05$, *** $p < 0.01$

Table S4. Analysis of Variance estimating the variation in stress response of a gene (as measured by its z-scores based on gene occurrence in environmental stress gradient) explained by the following factors in a linear regression: the stress category, the COG (Clusters of Orthologous Groups) category the gene belonged to, and the interaction between these two factors. Though mean z-scores varied between different stress variables and different COG categories, suggesting the average probability of being lost under stress varied by COG, the lack of interaction shows no evidence that some COG categories were more or less likely to be lost in different kinds of stress. COG category was derived from eggNOG-mapper output.

	ANOVA
Stress Variable	**F = 458.596*** **
	**p = 0.000**
COG Category	**F = 1.521*** **
	**p = 0.000**
Stress Variable × COG category	F = 0.856
	p = 0.971

* $p < 0.1$, ** $p < 0.05$, *** $p < 0.01$

Table S5: Patterns of purifying or positive selection in extant accessory genes, dN/dS, as predicted by the gene's z-score (stress response, based on gene occurrence in environmental stress gradient). The first column shows model results of the proportion of codons where dN/dS<1 within a gene (as determined by bayesian credibility), modelled using a beta distribution. Second column shows model results of the presence of any genes with at least one codon dN/dS>1, modelled using a binomial distribution. Both models show coefficients of effect sizes (*p*-values in brackets) relative to accessory genes that do not respond to stress (i.e. z-score ~ 0, which is the intercept). Significant positive coefficients indicate that genes which have a strong tendency of being lost in stress also have a significantly higher proportion of codons under purifying selection (dN/dS <1) and also have a significantly higher probability of containing any codon under positive selection (dN/dS credibly > 1). For the beta regression, the precision part of the model accounts for heterogeneity of variance (the precision is the inverse of the variance), which beta regressions permit.

	p(dN/dS < 1)	any(dN/dS > 1)
Intercept (No Change with Stress)	-0.082 (0.011)*	-3.127 (0.000)***
Negative Response to Stress	**1.166 (0.000)*** **	0.477 (0.018)*
Positive Response to Stress	**1.221 (0.000)*** **	0.283 (0.177)
Precision	**0.900 (0.000)*** **	
Error Family	Beta	Binomial
Num.Obs.	3000	3000
R^2 Pseudo	0.098	
AIC	-8131.4	1266.8
BIC	-8107.4	1284.8
Log.Lik.	4069.689	-630.398

* $p < 0.05$, ** $p < 0.01$, *** $p < 0.001$

Table S6: Mean change in codons under purifying selection in core genes as environmental stress increases (p-values in brackets). Because all genes had, on average, >99.9% of codons with dN/dS values between 0 and 1, the response variable is the mean proportion of codons dN/dS credibly less then 1 within a gene (i.e. % codons under purifying selection). Here, the mean proportion is calculated by averaging across all gene dN/dS proportion values within a single soil sample (n ~500 genes/soil sample). The coefficients in the table ('Mean') show effect sizes, where a significantly negative value indicates that the average proportion of codons < 1 is decreasing and indicate that core genes, on average, contain more synonymous ('neutral') SNP substitutions as stress increases. Neutral SNP are predicted to increase in frequency under weaker selection and/or stronger drift. The precision part of the model accounts for heterogeneity of variance (the precision is the inverse of the variance), which beta regressions permit.

	Mean	Precision
(Intercept)	1.966 (0.000)***	4.001 (0.000)***
Aridity	-0.104 (0.183)	-0.136 (0.451)
Acidity	0.159 (0.065)	0.570 (0.002)**
Heat	**-0.444 (0.000)*****	**-1.806 (0.000)*****
Salinity	**-0.561 (0.000)*****	**-1.320 (0.000)*****
Num.Obs.	60	60
R^2 Pseudo	0.322	0.322
AIC	-186.1	-186.1
BIC	-165.1	-165.1
Log.Lik.	103.033	103.033

* $p < 0.05$, ** $p < 0.01$, *** $p < 0.001$

Table S7. Change in environmentally stratified pairwise Fst values (computed from all core gene SNPs), as predicted by the four major environmental stress gradients. The regression coefficient from a multiple regression represents the change in average pairwise Fst computed among pairs of sequences from the same environmentally similar bin (see Supplementary Methods S6 for environmental binning procedure). A significant positive coefficient indicates that population differentiation increases as the mean environmental stress value increases. P-values shown in brackets are obtained from permutation tests. See Method H for full details on the calculation and statistical analyses.

	**Change in Fst**
Intercept	0.024
	(1.000)
Aridity	0.019
	(0.932)
Heat	**0.466****
	**(0.010)**
Salinity	**0.626*****
	**(0.000)**
Acidity	-0.073
	(0.703)
R^2	0.294

* $p < 0.1$, ** $p < 0.05$, *** $p < 0.01$

Supplementary Figures

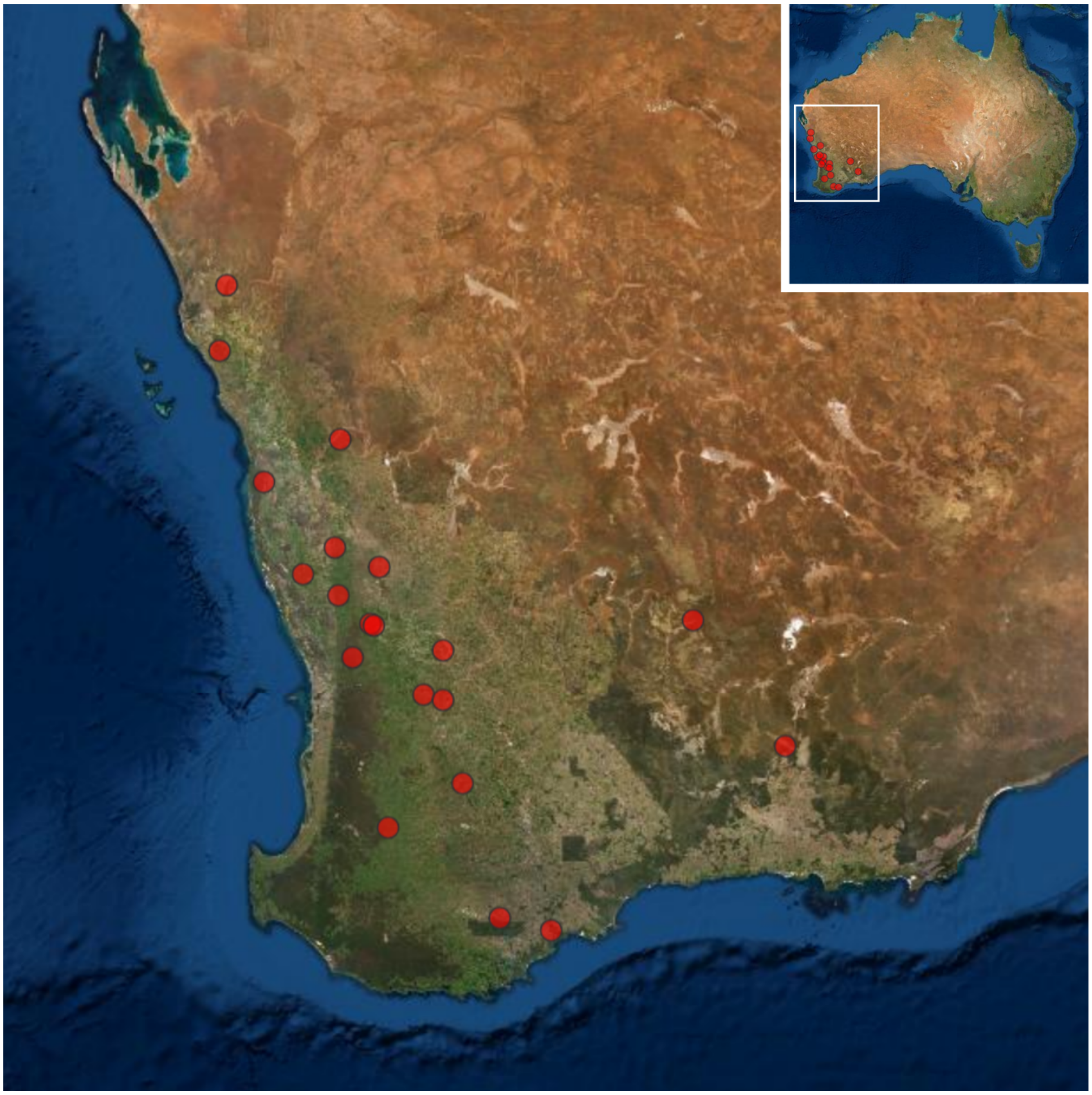

Figure S1: Locations of sampled sites in Australia

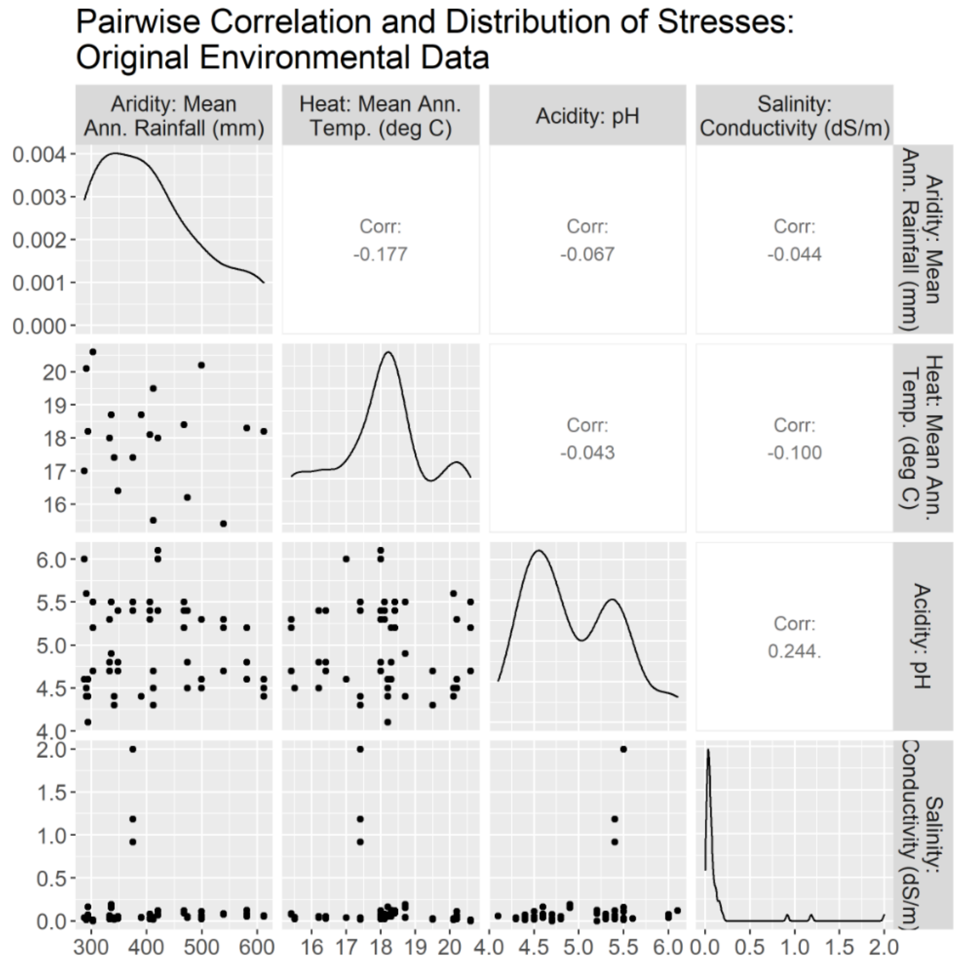
Pairwise Correlation and Distribution of Stresses:
Original Environmental Data
Aridity: Mean Ann. Rainfall (mm)
Heat: Mean Ann. Temp. (deg C)
Acidity: pH
Salinity: Conductivity (dS/m)
Corr: -0.177
Corr: -0.067
Corr: -0.044
Corr: -0.043
Corr: -0.100
Corr: 0.244.

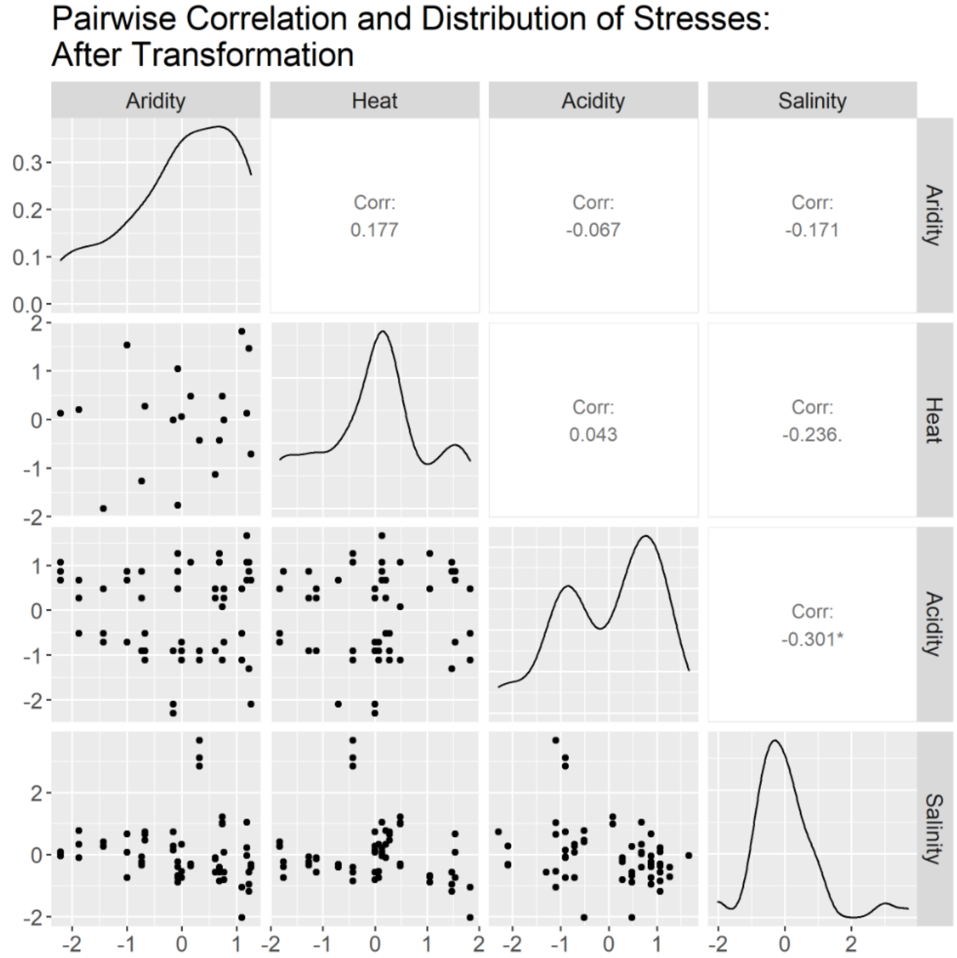
Pairwise Correlation and Distribution of Stresses:
After Transformation
Aridity
Heat
Acidity
Salinity
Corr: 0.177
Corr: -0.067
Corr: -0.171
Corr: 0.043
Corr: -0.236.
Corr: -0.301*

Figure S2: Summary of environmental stress gradients. Off-diagonals indicate correlation between transformed environmental factors and diagonals indicating data distribution of each transformed environmental factor. Strains were collected across 20 sites (3 soil samples/site). Climate associated stresses (heat and aridity) are estimated from Worldclim climate models at the site level (n=20). Soil chemistry factors (pH and salinity) were measured at the soil samples level (n=60). [*] indicates $p<0.05$ [.] indicates $p<0.10$

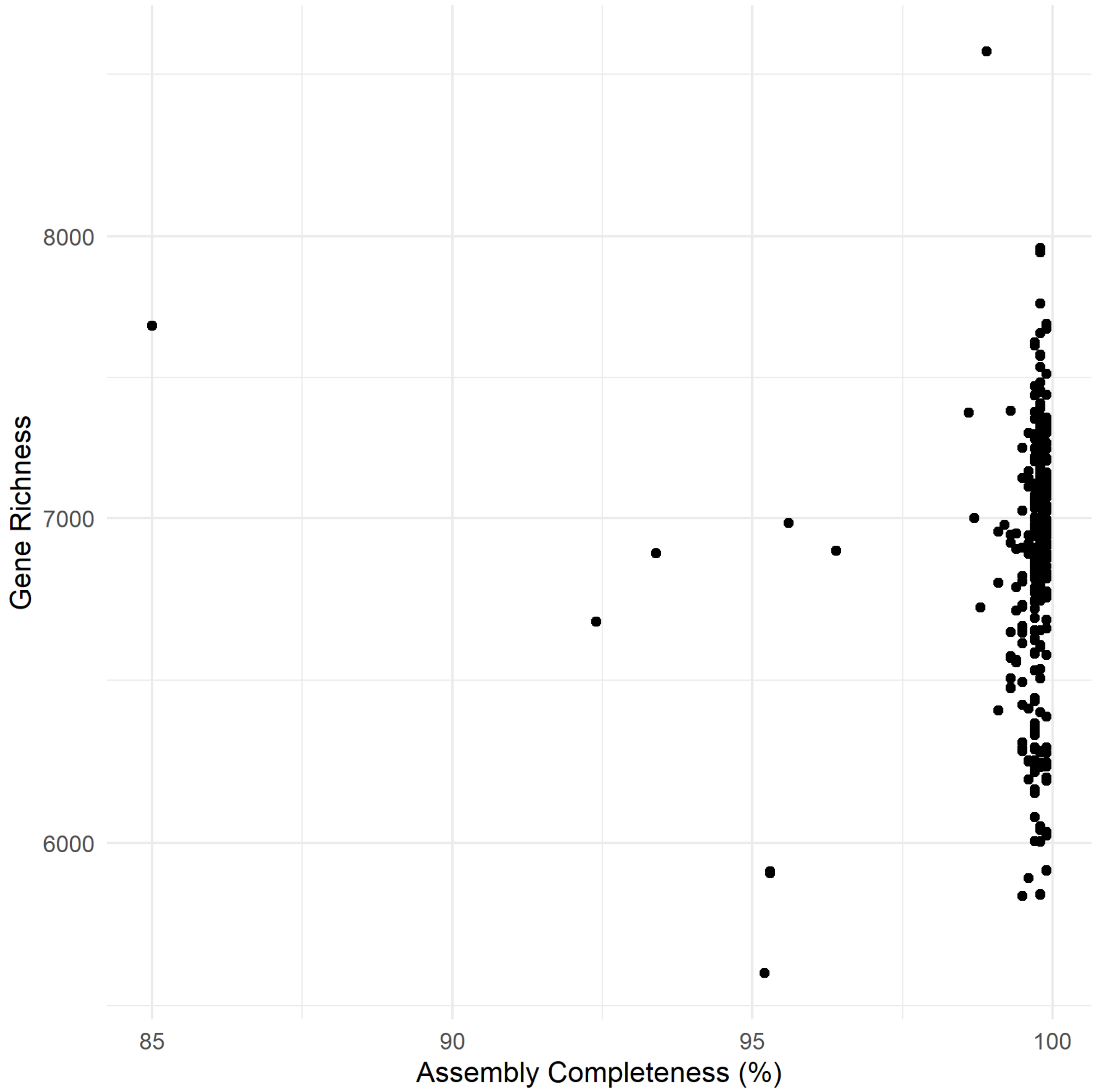

Figure S3. Relationship between gene richness and genome assembly completeness (calculated from BUSCO). $r = 0.042$, $p = 0.4224$.

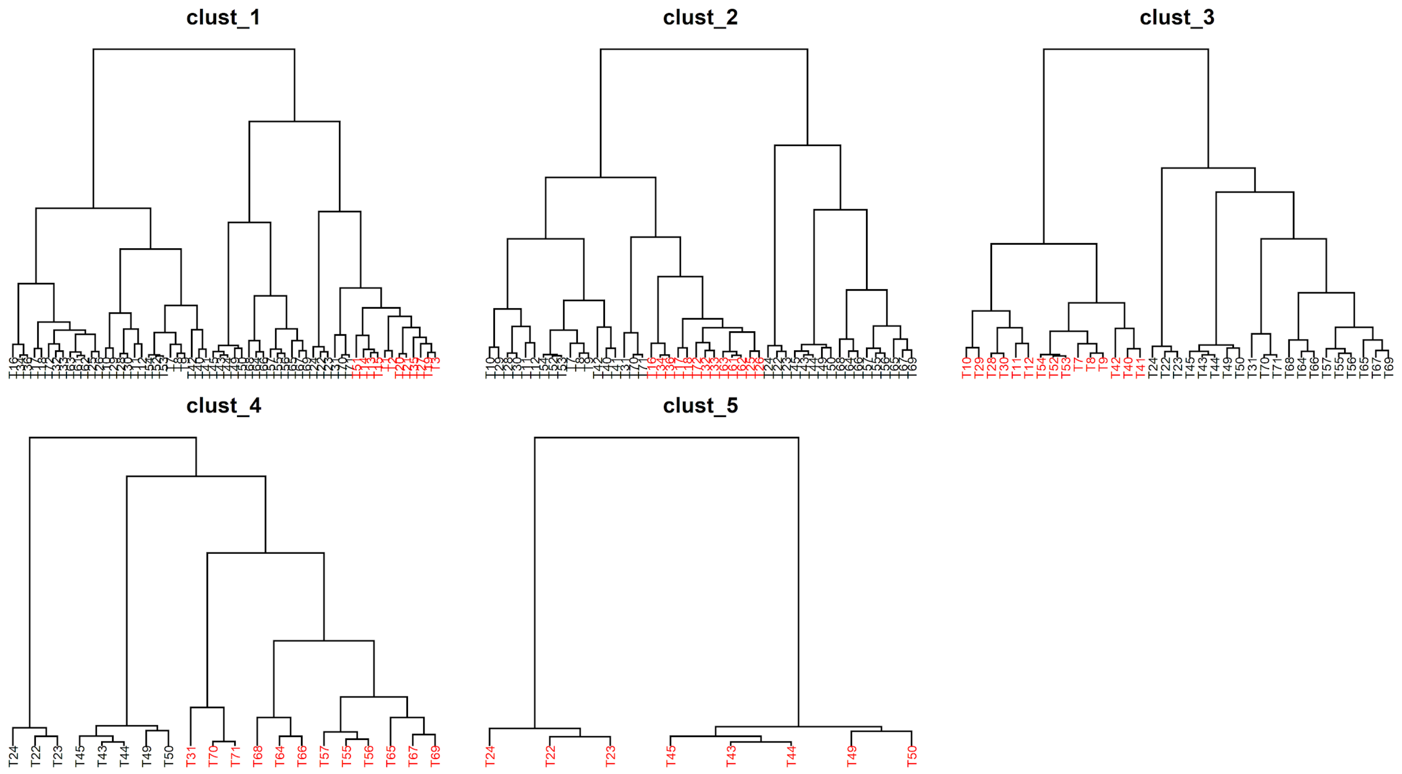

Figure S4: Figure demonstrating the iterative procedure for binning soil samples into 5 groups (see Supplementary Method S6 for full details). For each dendrogram, the red text indicates the clustered (i.e. binned) soil samples based on similarity in mean annual temperature, mean annual rainfall, soil pH and soil salinity.

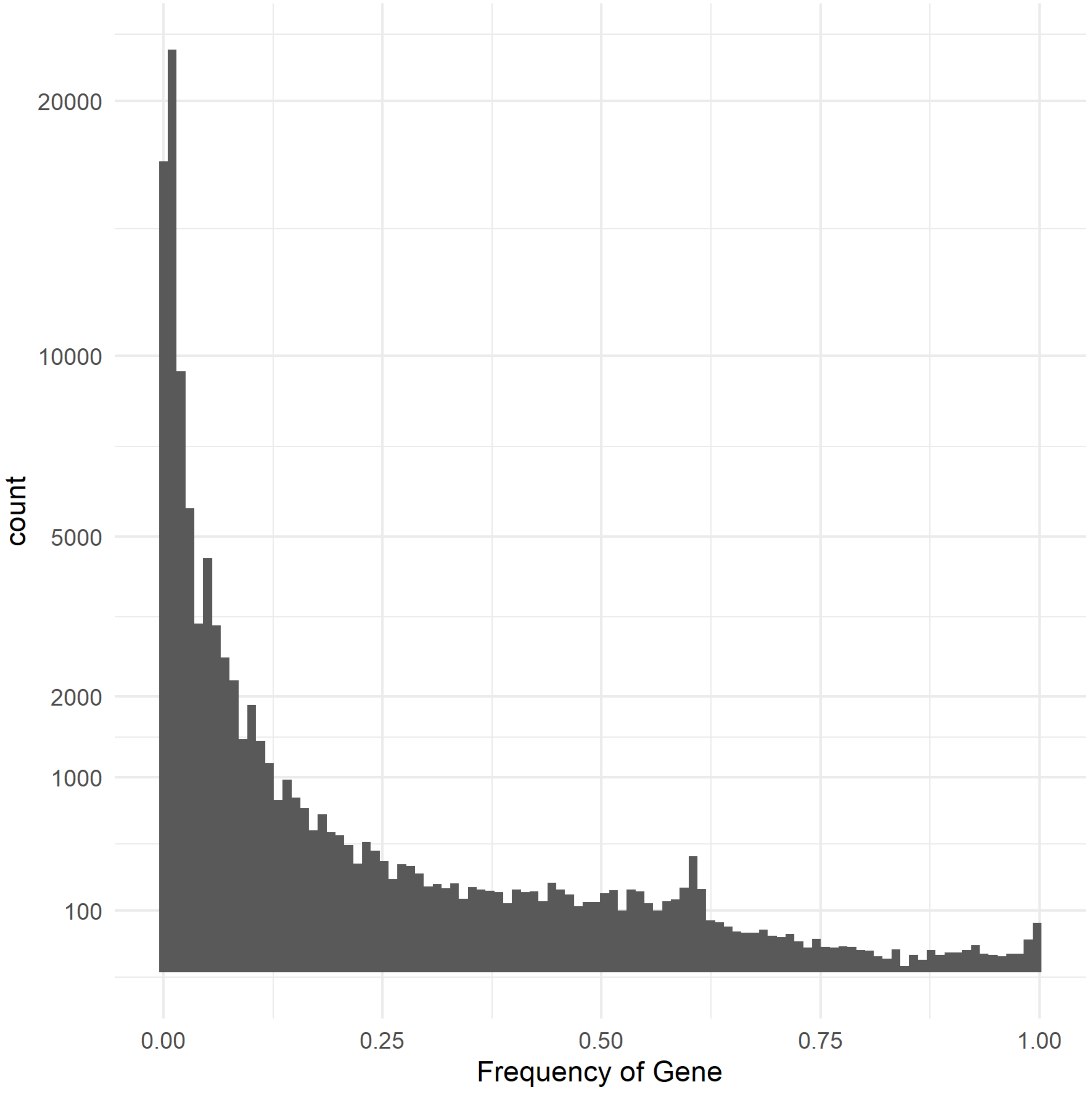

Figure S5: Flexible pangeome of *Bradyrhizobium* spp. Count indicates the number of unique protein coding genes. Frequency is calculated as the percentage of occurrence across 374 isolates.

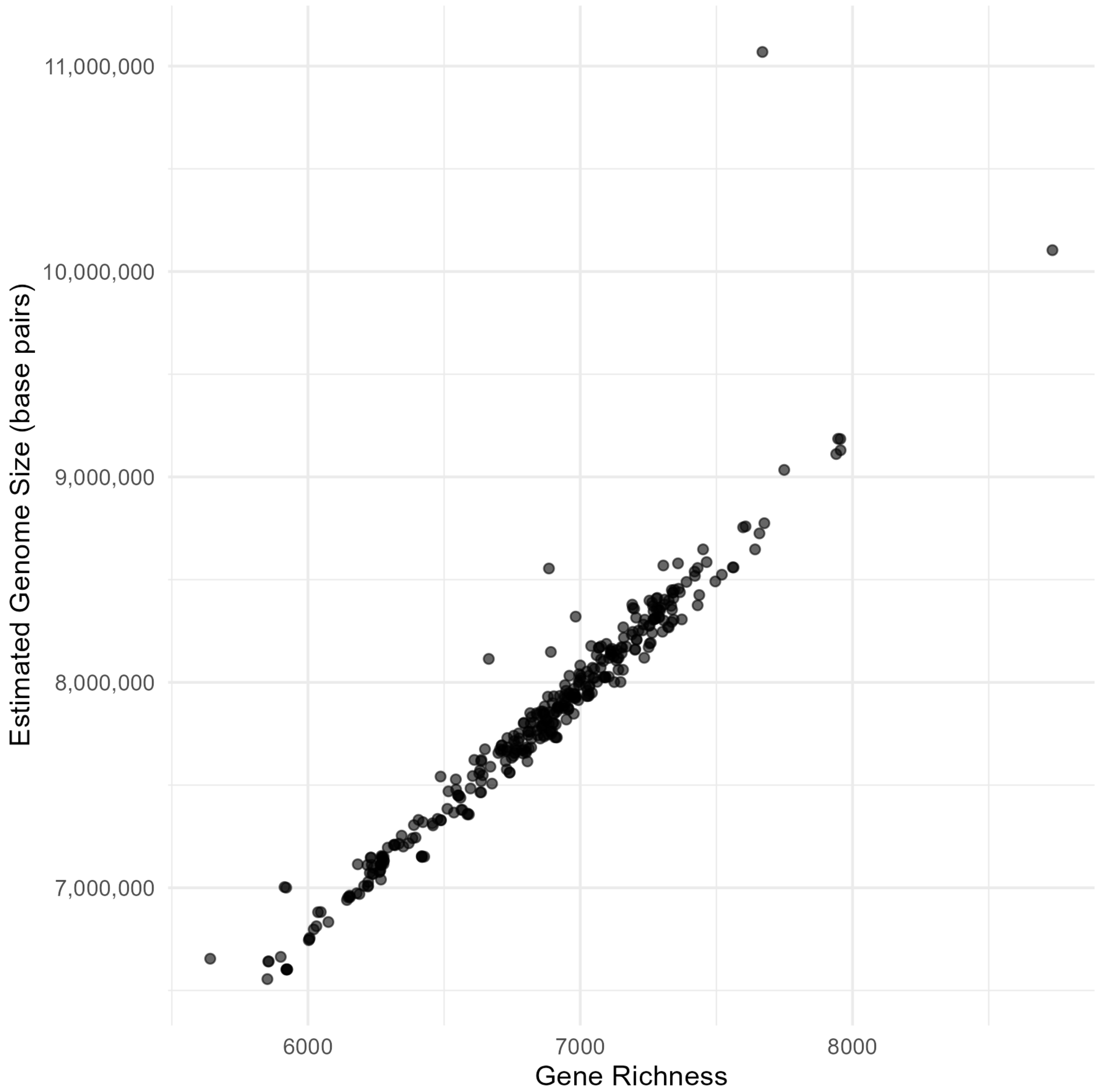

Figure S6: Correlation between gene richness (the number of genes per strain/genome) and the estimated genome size, measured as total genome length, in basepairs. Genome size was estimated based on total assembly length (in base pairs) and genome completeness scores obtained from BUSCO (i.e. draft-assembled genome length [in base pairs] / BUSCO score)

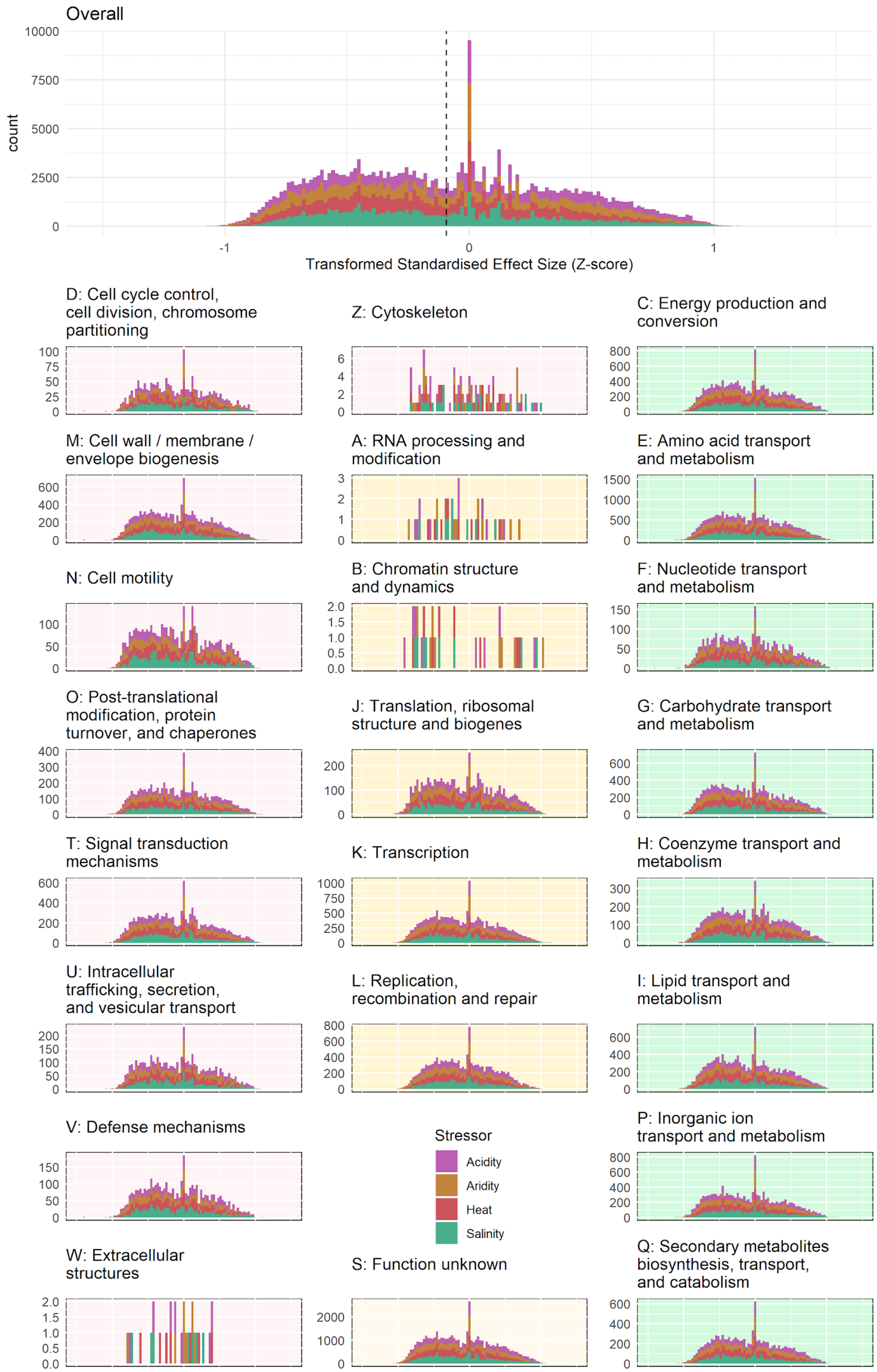

Figure S7: Histogram/density plot of z-scores derived from gene distribution models for each environment, according to COG function. See Table S4 for ANOVA results.

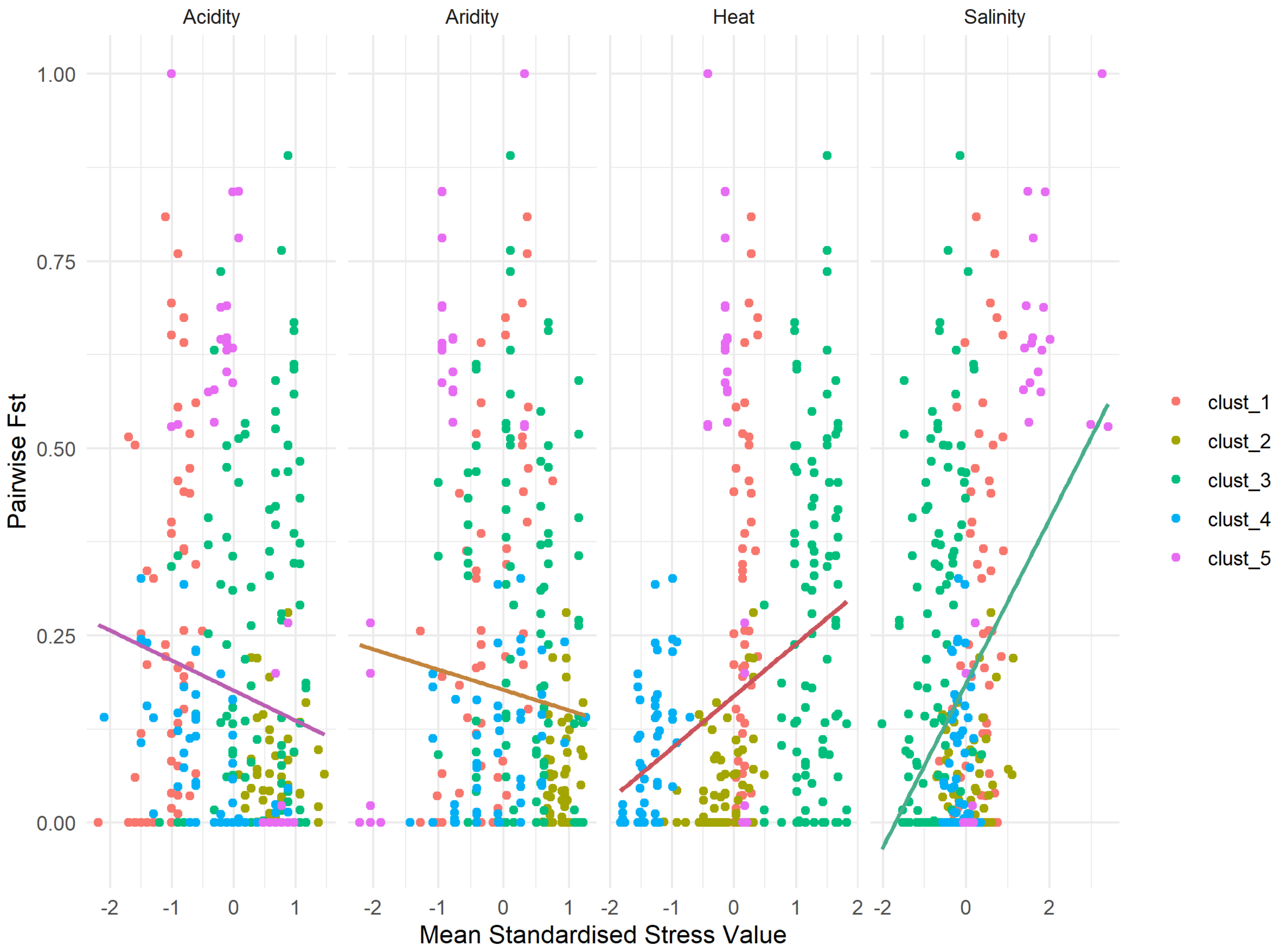

Figure S8: Environmentally stratified pairwise Fst values (computed from core gene SNPs), as predicted by four major environmental stress gradients in a linear multiple regression. Each cluster denotes a bin, where a bin refers to a grouping of soil samples that have more similar environmental characteristics (see Figure S4 for soil sample membership in each bin). Mean standardised stress value is calculated as the average environmental stress value between each pairwise comparison. The line of best fit is shown. See Supplementary Method S6 for full details on the binning algorithm used to group soil samples and Method H for environmentally stratified Fst calculation based on 5 soil bins (denoted by 'clust_'). Statistical model results are shown in Table S7.

References

1. Webber JBW. A bi-symmetric log transformation for wide-range data. *Meas Sci Technol* 2012; **24**: 027001.
2. Lynch M. Statistical inference on the mechanisms of genome evolution. *PLoS Genet* 2011; **7**: e1001389.
3. Lynch M, Bobay L-M, Catania F, Gout J-F, Rho M. The repatterning of eukaryotic genomes by random genetic drift. *Annu Rev Genomics Hum Genet* 2011; **12**: 347–366.
4. Wilson DJ, CRyPTIC Consortium. GenomegaMap: Within-Species Genome-Wide dN/dS Estimation from over 10,000 Genomes. *Mol Biol Evol* 2020; **37**: 2450–2460.
5. Grün B, Kosmidis I, Zeileis A. Extended Beta Regression in R: Shaken, Stirred, Mixed, and Partitioned. *Journal of Statistical Software, Articles* 2012; **48**: 1–25.
6. Ward JH. Hierarchical Grouping to Optimize an Objective Function. *null* 1963; **58**: 236–244.
7. Monlong J. Clustering into same size clusters. 2018. Github.
8. Ineichen R, Batschelet E. Genetic selection and de Finetti diagrams. *J Math Biol* 1975; **2**: 33–39.
9. Gibbs JW. Elementary Principles in Statistical Mechanics: Developed with Especial Reference to the Rational Foundation of Thermodynamics. 1902. C. Scribner's Sons.
10. Murdoch D, D. A, Nenadic O. Rgl: A r-library for 3d visualization with opengl. 2021.
11. Zhang D. A Coefficient of Determination for Generalized Linear Models. *Am Stat* 2017; **71**: 310–316.
12. Zhang D. rsq: R-Squared and Related Measures. 2021.
13. Chevan A, Sutherland M. Hierarchical Partitioning. *Am Stat* 1991; **45**: 90–96.
